# Multifunctional Lanthanide-Based Metal–Organic Frameworks Derived from 3-Amino-4-hydroxybenzoate: Single-Molecule Magnet Behavior, Luminescent Properties for Thermometry, and CO_2_ Adsorptive Capacity

**DOI:** 10.1021/acs.inorgchem.2c00544

**Published:** 2022-08-08

**Authors:** Estitxu Echenique-Errandonea, Ricardo F. Mendes, Flávio Figueira, Duane Choquesillo-Lazarte, Garikoitz Beobide, Javier Cepeda, Duarte Ananias, Antonio Rodríguez-Diéguez, Filipe A. Almeida Paz, José M. Seco

**Affiliations:** † Departamento de Química Aplicada, Facultad de Química, Universidad del País Vasco UPV/EHU, Paseo Manuel Lardizabal, No 3, 20018 Donostia-San Sebastián, Spain; ‡ Department of Chemistry, CICECO—Aveiro Institute of Materials, University of Aveiro, 3810-193 Aveiro, Portugal; § Laboratorio de Estudios Cristalográficos, IACT, CSIC-UGR, Av. Las Palmeras no 4, 18100 Granada, Spain; ∥ BCMaterials, Basque Center for Materials, Applications and Nanostructures, UPV/EHU Science Park, 48940 Leioa, Spain; ⊥ Departamento de Química Inorgánica, Facultad de Ciencias, Universidad de Granada, Av. Fuentenueva S/N, 18071 Granada, Spain; ∇ Departamento de Química Orgánica e Inorgánica, Universidad del País Vasco UPV/EHU, 48940 Leioa, Spain

## Abstract

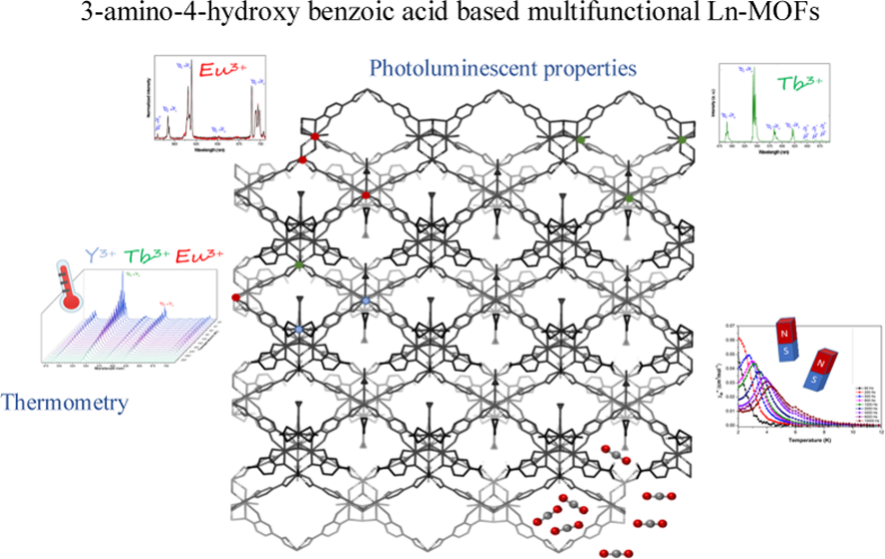

Herein, we describe and study a new family of isostructural multifunctional metal–organic frameworks (MOFs) with the formula {[Ln_5_L_6_(OH)_3_(DMF)_3_]·5H_2_O}*_n_* (where (H_2_L) is 3-amino-4-hydroxybenzoic acid ligand) for magnetism and photoluminescence. Interestingly, three of the materials (Dy-, Er-, and Yb-based MOFs) present single-molecule magnet (SMM) behavior derived from the magnetic anisotropy of the lanthanide ions as a consequence of the adequate electronic distribution of the coordination environment. Additionally, photoluminescence properties of the ligand in combination with Eu and Tb counterparts were studied, including the heterometallic Eu–Tb mixed MOF that shows potential as ratiometric luminescent thermometers. Finally, the porous nature of the framework allowed showing the CO_2_ sorption capacity.

## Abstract 2

A new family of isostructural multifunctional metal−organic frameworks has been described and studied for magnetism and photoluminescence. Interestingly, some materials present single-molecule magnet behavior, and photoluminescence properties of the ligand in combination with Eu and Tb counterparts were studied, including the heterometallic Eu−Tb mixed MOF that shows potential as ratiometric luminescent thermometers. Finally, the porous nature of the framework allowed showing the CO_2_ sorption capacity.

## Introduction

Metal–organic frameworks (MOFs) are organic–inorganic hybrid materials self-assembled by metal ions/clusters with organic linkers through metal–organic linker coordination bonds. As metal ions/clusters generally display certain preferred coordination geometries, self-assembly of these moieties (known as nodes) with organic ligands (linkers) of predetermined shapes and predictable coordination patterns can give rise to rationally designed MOFs with anticipated structures.^1^ These materials are also well known for their permanent porosity with a significantly high surface area, which makes them very promising for applications related to gas capture.^2,3^ In this regard, pore surface’s tunability by pre- or postsynthetic modifications permit convenient optimization and maximization of the pore size and shape to fully exploit pore space for selective adsorption and storage. Moreover, the crystallinity of these materials allows the precise analysis of adsorption sites, which helps us to understand that the magnitude of adsorbate–adsorbent interactions is within the pores.^4^ In particular, lanthanide-based MOFs (Ln–MOFs) are a class of crystalline materials that have attracted great attention during the last decades due to their intrinsic advantages such as coordination versatility and broad application spectrum owing to their unique properties based on f-electrons.^5^ In fact, lanthanide ions offer the possibility to incorporate both luminescent centers and magnetic properties in a single material, enabling combinations that are ideally shaped for a particular application, while the material is imbued with a multifunctional character.^6−9^

Lanthanide’s electrons are located in 4f orbitals, which are shielded by occupied 5s^2^ and 5p^6^ orbitals from the ligand field. Because of this fact, the coordination environment around the 4f ion remains almost undisturbed, giving rise to high spin–orbit coupling interactions. Therefore, when describing the magnetic properties of lanthanide ions, it is necessary to consider a spin–orbit coupling term, which is described by the *M*_J_ quantum number. Overall, the rational design of materials with single-molecule magnet (SMM) behavior requires, in addition to a well-defined ground state with the highest *M*_J_ value, sizeable energy separation with the excited *M*_*J±1*_ sublevels to get high energy barriers (*U*_eff_) for the reversal of magnetization, and thus high-performance SMMs. This energy barrier is dependent on a parameter called anisotropy, which is an intrinsic characteristic of lanthanide ions and their coordination spheres. The electronic cloud of each lanthanide ion disposes a particular shape in each *M*_J_ level, although, when stabilizing the ground state *M*_J_ sublevel, two main shapes are distinguished: oblate and prolate.^10^ To enhance magnetic anisotropy in the ground state *M*_J_ sublevel, it is important to suitably select the coordination environment of the metal, i.e., the ligand distribution around the metal center (in other words the ligand field), to favor anisotropic electron density of the lanthanide ion, and thus, rationally design materials with greater energy barriers. In this regard, according to the lanthanide ions’ anisotropic electron density—leaving apart Gd^3+^, which is isotropic—, oblate ions (e.g., Dy^3+^ and Tb^3+^ ions) should possess ligand donor atoms with the greatest electron density coordinated at the axial positions, whereas prolate ions (e.g., Er^3+^ and Yb^3+^) acknowledge the coordination of ligand donor atoms with greatest electron density coordinated at the equatorial positions to maximize the anisotropy of the metal center.^10^

Apart from exploiting magnetic properties, lanthanide ions offer interesting photoluminescence properties characterized by emissions that cover a vast range of the electromagnetic spectrum. Even if they show small absorptive coefficients, each lanthanide ion shows characteristic hypersensitive and narrow emissive lines, converting these metals particularly suitable for the elaboration of light-emitting devices.^11^ In the particular case of lanthanide-based MOFs, the photoluminescence properties arise from both the metal center and the organic ligand, which makes the aforementioned structural design of high importance to modulate the emission to a specific application such as sensing, diodes, display technology, etc.^12,13^ Notably, among aforementioned applications, in recent years, much effort has been devoted to the development of Ln^3+^ ratiometric thermometers.^14^ Compared to conventional contact thermometry, luminescent thermometry exhibits a noninvasive and robust technique with faster response, higher accuracy, and spatial resolution where generally conventional thermometry lacks effectiveness.^15^ In general, the absolute temperature in lanthanide-based luminescent thermometers is optically determined, preferably, via the intensity ratio of two Ln^3+^ emitting centers. Often, Eu–Tb mixed MOFs are presented as good candidates where the intensity ratio of the ^5^D_4_ → ^7^F_5_ and of the ^5^D_0_ → ^7^F_2_ transitions of Tb^3+^ and Eu^3+^, respectively, are compared.^15^ It must be noted that since 2012, when the first example of a ratiometric Eu–Tb mixed MOF luminescent thermometer was described,^16^ the number of these materials has potentially increased. So far, the reported luminescent thermometers display greater thermal sensitivity for a specific temperature range and they are, hence, classified among the temperature region in which they can perform. In this way, thermometers performing in the cryogenic region (<100 K), in medium (100–300 K), in biological (298–323 K), and (>400 K) in high-temperature domains may be distinguished. In particular, thermometers performing in the cryogenic region are in great demand since they can find application in fields of superconducting magnets, aerospace, and nuclear fusion power.^17−19^ However, at present, the number of lanthanide-based MOF thermometers covering the cryogenic range is still very limited.^20−22^

As previously stated, MOF’s porosity and high surface areas enable their application in gas adsorption/separation processes. Among other gases, the current increase in atmospheric CO_2_ concentration levels, resulting from the combustion of fossil fuels, is nowadays a worldwide environmental concern. Until now, great efforts are being done to develop new methodologies and technologies to effectively capture this gas to mitigate its emission into the atmosphere.^23,24^ While still at its infancy, a significant progress has been made in the development of MOFs for CO_2_ capture in recent years. Nonetheless, the implementation of MOFs as CO_2_ adsorbents is still a challenging matter.^4^ Four main mechanisms rule the selective adsorption of CO_2_ using MOFs as adsorbents: (1) the size and shape exclusion, only molecules with a specific shape and below certain size could only break through the pore; this effect is called the molecular sieving effect. (2) The interaction between the pore surface and the adsorbate, (3) the control over the pore size of the adsorbent and the kinetic diameter of two molecules required to be separated, and (4) the diffusion speed of guest molecules and compatibility of the pore diameter will determine the selective-adsorption process.^7,25^ Additionally, it must be noted that for MOFs being used as CO_2_ adsorbents before the adsorption process, an activation may be required via applying vacuum and/or high temperatures with the goal of removing coordinated and crystallization solvent molecules to give rise to open metal sites or coordinatively unsaturated sites (*cus*) with which guest molecule will interact. The strength of this interaction is defied by the heat of enthalpy or isosteric heat (*Q*_st_) and describes the affinity of the MOFs to adsorb CO_2_.^4^ Generally, material activation promotes an uptake of guest molecules, CO_2_ in this case, improving by far the adsorption capacity of the MOFs.

Within this framework, the quest for multifunctional magneto-luminescent porous molecule-based materials holding a set of properties is a field of demanding interest. Many examples have been reported so far;^8,9,26−28^ in this regard, our research group has paid attention to the preparation of Ln–MOFs for adsorption processes and focused on designing novel multifunctional metal–organic frameworks exhibiting magnetic and luminescence properties. In this work, we report on a novel family of isostructural porous compounds formulated as {[Ln_5_L_6_(OH)_3_(DMF)_3_]·5H_2_O}*_n_* based on a 3-amino-4-hydroxybenzoic acid ligand (H_2_L).^29^ In addition to their synthesis and physicochemical characterization, magnetic properties have been accomplished based on samples containing oblate and prolate lanthanide(III) ions as well as on magnetically diluted materials. Moreover, photoluminescence properties of the ligand and Tb^3+^ and Eu^3+^ samples and the performance as ratiometric thermometers in heterometallic materials have been studied. Finally, the adsorptive capacity of the three-dimensional (3D) Ln–MOFs to adsorb CO_2_ has also been analyzed.

## Materials and Methods

### Preparation of Complexes

All chemicals were of reagent grade and were used as commercially obtained without further purification. For all tested synthetic methods, neodymium(III) nitrate hydrate (at least 99.9% of purity, Alfa Aesar), samarium(III) nitrate hexahydrate (at least 99.9% of purity, Stem Chemicals), europium(III) nitrate hexahydrate (99.9%, Alfa Aesar), gadolinium(III) nitrate hexahydrate (99.9%, Aldrich), terbium(III) nitrate hydrate (99.9%, Alfa Aesar), dysprosium nitrate hydrate (99.9%, Aldrich), holmium(III) nitrate hydrate (99.9%, Alfa Aesar), erbium(III) nitrate hydrate (99.9%, Stem Chemicals), thulium(III) nitrate hexahydrate (99.9%, Stem Chemicals), and yttrium(III) nitrate hexahydrate (99.9% purity, Fluorochem) were employed as metallic precursors. 3-Amino-4-hydroxybenzoic acid ligand (H_3_L, C_7_H_7_NO_3_, 97% of purity) was purchased from Fluorochem.

### Synthesis of {[Ln_5_L_6_(OH)_3_(DMF)_3_]·5H_2_O}*_n_*

#### General Procedure for the Synthesis of Single Crystals

A total of 0.01 g (0.0625 mmol) of a 3-amino-4-hydroxybenzoic acid organic linker was dissolved in 0.5 mL of dimethylformamide (DMF) containing 10 μL of Et_3_N (0.072 mmol). Then, 0.0434 mmol of the corresponding lanthanide nitrate salt was dissolved into 0.5 mL of distilled water in a separate vial. Once dissolved, 0.5 mL of H_2_O was added to the ligand solution and 0.5 mL of DMF to the metal solution. The metal solution was added dropwise to the ligand solution under magnetic stirring. The resulting brownish-yellow solution was poured into a screw-capped vial (6 mL) and introduced into an oven at 100 °C for 2 h to give rise to hexagonal-shaped single crystals. Single-crystal X-ray structure determination, elemental analysis (EA), and thermogravimetric analysis (TGA) confirm the general formula {[Ln_5_L_6_(OH)_3_(DMF)_3_]·5H_2_O}*_n_*. Detailed information is given in the Supporting Information.

#### General Procedure for the Scaled-Up Synthesis

Further, 0.2 g (1.2 mmol) of a 3-amino-4-hydroxybenzoic acid ligand was weighed and dissolved in 3 mL/2 mL of a DMF/H_2_O solvent mixture containing 200 μl of Et_3_N (1.44 mmol). To this solution, a solution containing 0.868 mmol of Ln(NO_3_)_3_·nH_2_O, dissolved in 1 mL of water, was added dropwise. A precipitate seems to be formed in the beginning though it is eventually redissolved to give rise to a brownish-yellow solution. This solution was placed in a microwave and heated at 100 °C for 2 h to get around 60–85 mg of Ln–MOFs (yielding ∼18–26%). The purity of the product was confirmed by powder X-ray diffraction (PXRD).

#### General Procedure for the Synthesis of Heterometallic Materials

##### Synthesis of Heterometallic Materials for Magnetic Dilution

A total of 0.100 g (0.625 mmol) of a 3-amino-4-hydroxybenzoic acid organic linker was dissolved in 3 mL/2 mL of a DMF/H_2_O solvent mixture containing 100 μL of Et_3_N (0.720 mmol). To this solution, a solution containing 0.434 mmol of the corresponding salt mixture dissolved in 1 mL of water was added dropwise. For the synthesis of magnetically diluted samples, yttrium was employed as a diamagnetic metallic center. Three heterometallic Y^3+^–Ln^3+^ compounds were prepared where Ln^3+^ = Dy^3+^, Er^3+^, or Yb^3+^ with 30:1% Y^3+^/Ln^3+^ doping proportion. The metal mixture solution was added dropwise to the ligand solution under magnetic stirring. The resulting brownish-yellow solution was placed in a microwave and heated at 100 °C for 2 h. The purity of the product was confirmed by PXRD.

##### Heterometallic Materials Tested in Radiometric Thermometry

A total of 0.1 g (0.625 mmol) of a 3-amino-4-hydroxybenzoic acid organic linker was dissolved in 3 mL/2 mL of a DMF/H_2_O solvent mixture containing 100 μL of Et_3_N (0.720 mmol). To this solution, a solution containing 0.434 mmol of the corresponding lanthanide salt mixture dissolved in 1 mL of water was added dropwise. Accordingly, three additional heterometallic compounds with Y^3+^ or Gd^3+^ and Tb^3+^/Eu^3+^ mixed lanthanide ions were prepared with the following doping proportions Y^3+^/Tb^3+^/Eu^3+^ 50:45:5% and 50:40:10% and Gd^3+^/Tb^3+^/Eu^3+^ 50:40:10%. The heterometal solution was added dropwise to the ligand solution under magnetic stirring. The resulting brownish-yellow solution was placed in a microwave and heated at 100 °C for 2 h. The purity of the product was confirmed by PXRD.

## Results and Discussion

### Crystal Structure Details

Single-crystal X-ray crystallographic studies on {[Ln_5_L_6_(OH)_3_(DMF)_3_]·5H_2_O}*_n_* (where Ln = **1**_**Nd**_, **2**_**Sm**_, **3**_**Eu**_, **4**_**Gd**_, **5**_**Tb**_, **6**_**Dy**_, **7**_**Ho**_, **8**_**Er**_, **9**_**Tm**_ and **10**_**Yb**_) reveal that three-dimensional (3D) lanthanide MOFs **1**–**10** crystallize in the hexagonal *P6*_*3*_*/m* space group (for details, see Table S2). Furthermore, X-ray crystallographic analysis suggested that **1**–**10** are isostructural compounds. So, in the following section, we will only discuss the structure of **6**_**Dy**_ in detail as a representative example of the family. The asymmetric unit of **6**_**Dy**_ is comprised of two crystallographically independent Dy^3+^ ions and a deprotonated ligand (L^2–^), as well as a coordinated DMF solvent-molecule. The organic linker, 3-amino-4-hydroxybenzoic acid, coordinates to the lanthanide ion by the carboxylate moiety in a bidentate way, along with the hydroxyl and amino groups in a monodentate way.

Dy1/Dy2 atoms present nine-coordinated and eight-coordinated environments, respectively. Dy1 exhibits a DyN_3_O_6_ environment composed of the coordination of three nitrogen atoms and three oxygen atoms belonging to amino and hydroxy groups of the organic ligand, whereas the remaining three oxygen atoms pertain to hydroxyl anions. Instead, Dy2 completes the coordination shell by six donor atoms belonging to the carboxylate moiety of the ligand in addition to the hydroxyl anion and a DMF solvent molecule. Continuous shape measurements (CShMs)^30^ reveal that Dy1 and Dy2 atoms build different polyhedra, with that of Dy1 resembling a spherical tricapped trigonal prism (TCTPR-9) and that of Dy2 a triangular dodecahedron (TDD-8, see Tables S5 and S6 for more detailed information).

**Figure 1:**
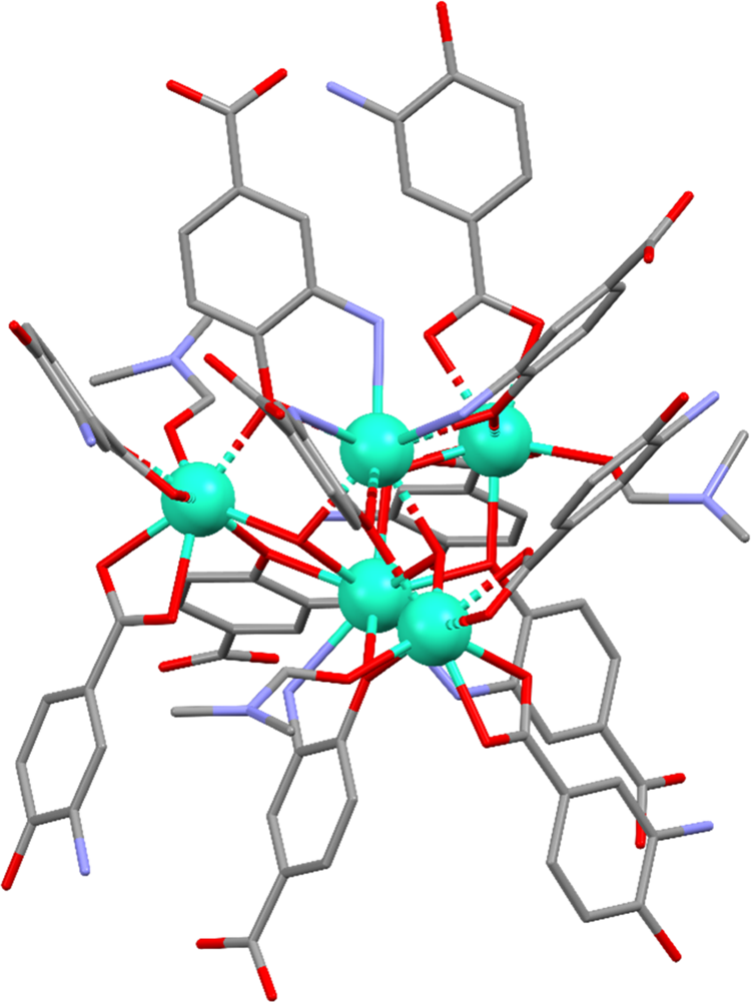
Excerpt of the coordination mode of the 3-amino-4-hydroxybenzoic acid ligand among the pentanuclear node; hydrogen atoms have been omitted for the sake of clarity.

Both coordination environments are interconnected by hydroxyl anions, which act as μ_3_-*O* to give rise to Ln_5_(OH)_3_ clusters (Figure 1), which can be considered the secondary building unit (SBUs) of the structure. Each of these clusters is connected to six neighboring SBUs in such a way that it may be referred to as a six-connected node. The analysis of the topology by means of TOPOS Pro software^31^ reveals that **6**_**Dy**_ presents a 6-connected uninodal net with the (4^9^·6^6^) point symbol and **acs** topology. Looking at the structure along the *c* axis, one can see narrow microchannels that correspond to 19.3% of the unit-cell volume according to the geometrical calculations performed with the PLATON-v1.18 program.^32^ Additionally, the porous nature of this MOF forces it to crystallize with several solvent molecules within the pores, which account for five crystallization water molecules according to the thermogravimetric analysis.

Compounds **1**–**10** are also isotypic to an yttrium-based MOF previously reported by our group.^29^ Even though, Ln1 and Ln2 coordination environments in compounds **1**–**10** are exactly the same as the yttrium-based MOF, in advance compound **11**, single-crystal parameters of the latter diamagnetic counterpart differ slightly, a reason why powder X-ray diffraction pattern shows slightly shifted diffraction maxima that is probably caused by the smaller size of the yttrium(III) ion compared to lanthanide(III) ions (see Figure S3). At this point, it is worth highlighting that depending on the Y^3+^ to Ln^3+^ doping proportion, the PXRD patterns present diffraction maxima corresponding to both pure compounds **11** and **1**–**10** (see Figure S5). Nevertheless, the occurrence of phase segregation has been discarded by SEM mapping experiments, which clearly confirm the random distribution of the three elements along a single crystal (for more details, see Figure S24).

In this work, the yttrium-based material gives us the opportunity to get a deeper insight into both magnetic and luminescence properties of the Ln–MOF family. On the one hand, its diamagnetic nature allows us to magnetically dilute paramagnetic centers to further examine the slow relaxation processes of single ions. On the other hand, a Y-based compound can be used as a matrix to be doped with Eu^3+^ and Tb^3+^ ions to modulate the emission signal and evaluate their possible application in ratiometric thermometers.

### Magnetic Properties

#### DC Magnetic Properties

**Figure 2:**
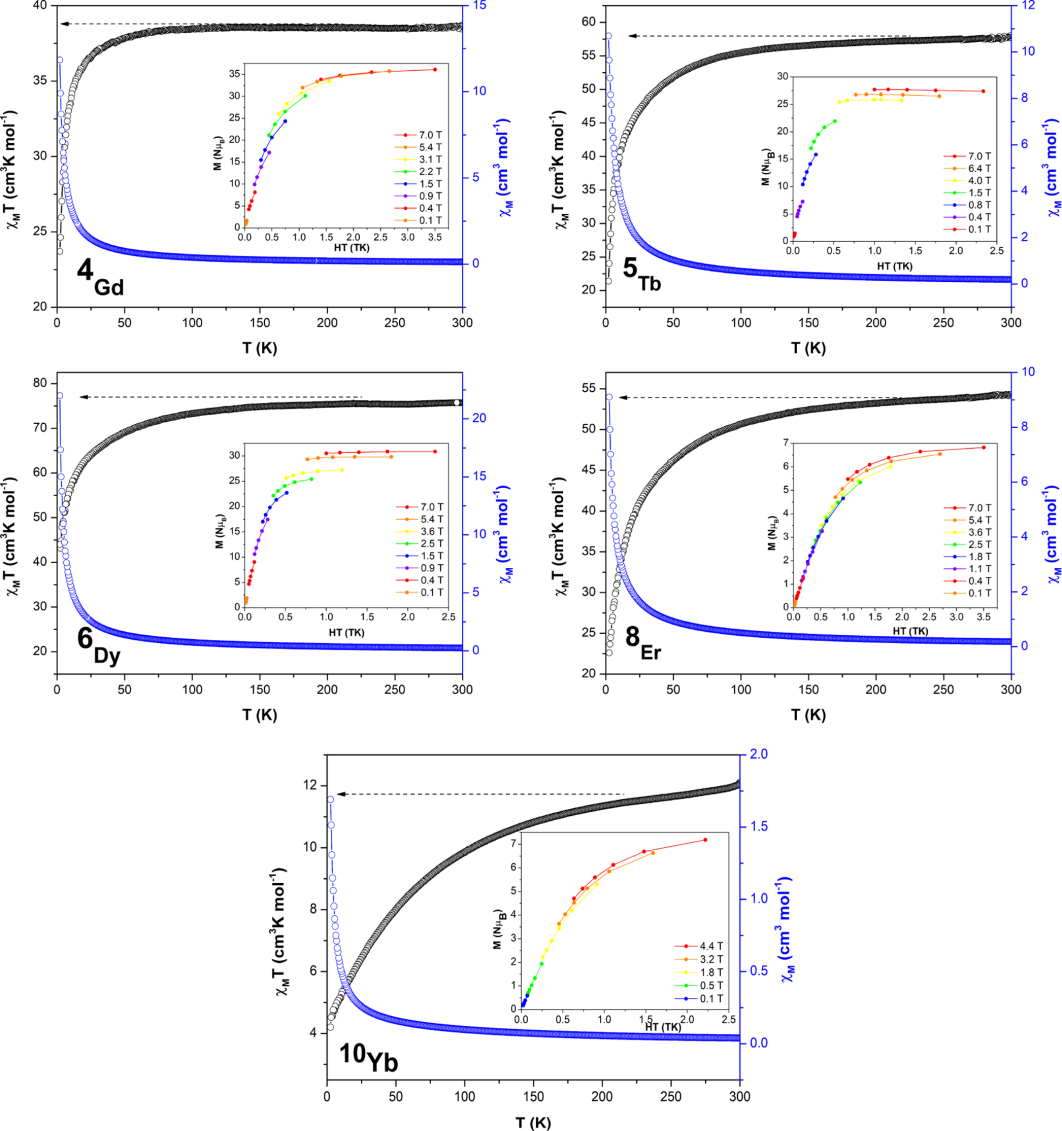
Temperature dependence of the χ_M_*T* product at 1000 Oe for complexes 4_Gd_–6_Dy_, 8_Er_, and 10_Yb_. Inset: isothermal reduced magnetization curves in the 2–7 K temperature range. Solid lines are a guide to the eye.

Magnetic molar susceptibility (χ_M_) measurements were acquired on polycrystalline samples in compounds **4**–**6**, **9**, and **10** in the temperature range of 2–300 K. The obtained room-temperature χ_M_*T* values are very close to the theoretical values expected for five free noninteracting ions considering a regular population of the Stark sublevels in their ground states (χ*T*_free ion_ 40, 60, 71, 58, and 13 cm^3^ K mol^–1^ for Gd^3+^(**4**), Tb^3+^(**5**), Dy^3+^(**6**), Er^3+^(**9**), and Yb^3+^ (**10**), respectively, Figure 2). Cooling down the samples, the χ_M_*T* product shows a progressive drop up to 50 K and a sharp drop at low temperature in compounds **4**_**Gd**_, **5**_**Tb**_, **6**_**Dy**_, and **9**_**Er**_. On its part, compound **10**_**Yb**_ shows a much slower decay to values of 4.20 cm^3^ K mol^–1^ at 2.0 K. Although the general trend of the χ_M_*T* product may be associated to the thermal depopulation for the excited *M*_J_ sublevels derived from the crystal-field splitting of the corresponding ground term of the Ln^3+^ ions (^8^S_0_, ^7^F_6_, ^6^H_15/2_, ^5^I_8_, and ^2^F_7/2_, respectively, for **4**_**Gd**_, **5**_**Tb**_, **6**_**Dy**_, **9**_**Er**_, and **10**_**Yb**_), the decrease of the χ_M_*T* product in compound **4**_**Gd**_ indicates that the occurrence of antiferromagnetic interactions must not be discarded. The field dependence of the magnetization was investigated in the range of 0–7 T at a 2–7 K temperature range. Isothermal reduced magnetization curves (insets) display saturation for compound **4**_**Gd**_ reaching a maximum of 35 NμB, which comes in line with the value expected for the pentanuclear node. The saturation in reduced magnetization curves and the superimposition in *M* vs *H*/*T* plots is an expected feature due to the isotropic nature of a Gd^3+^ ion. Instead, these curves (insets) lack of saturation (far from the expected saturation values of 9, 10, 9, and 4 NμB expected for one Tb^3+^, Dy^3+^ Er^3+^, and Yb^3+^ ion, respectively) for the rest of compounds, which in addition to the nonsuperimposition of the *M* vs *H*/*T* plots corroborates the presence of magnetic anisotropy in compounds **5**_**Tb**_, **6**_**Dy**_, **9**_**Er**_, and **10**_**Yb**_.

#### AC Magnetic Properties

Dynamic ac magnetic susceptibility measurements were performed in compounds **6**_**Dy**_, **8**_**Er**_, and **10**_**Yb**_. We carefully selected these materials as they show different shapes of free-ion electron density. Ln^3+^ ions with anisotropic electronic density are classified into two groups: oblate and prolate. Dy^3+^ is an oblate-type ion; so, to enhance its anisotropy, the ligand donor atoms with greatest electron density should coordinate at axial positions, whereas Er^3+^ and Yb^3+^ ions are prolate, indicating that the largest axial anisotropy is obtained by equatorial ligand coordination. In an attempt to understand which type of ion would better suit to present SMM behavior in this particular system consisting of pentanuclear clusters, both type of ions were studied. Dy^3+^-based SMMs are among the most prolific examples among lanthanide clusters with interesting single-molecule magnet properties, in contrast to Yb^3+^ analogues, which are very scarce.^33,34^ One of the main reasons is the shape of free-ion electron density. Moreover, the ligand field seems to cause a deeper effect in breaking the degeneration of *M*_J_ sublevels for a Dy^3+^ ion than for Er^3+^ and Yb^3+^ ions, in such a way that the former often presents greater magnetic anisotropy.^35^

Ac measurements under an alternating field of 3.5 Oe reveal that none of the compounds exhibit frequency-dependent signals above 2 K under a zero applied dc field. This effect can be connected to the relaxation of the magnetization via quantum tunneling (QTM),^36^ (in other words, bypassing between degenerated energy levels not needing to overcome the thermal energy barrier) and to the relatively weak coupling interactions among 4f ions, which lead to weak interactions that could facilitate the fast relaxation of the magnetization hiding the SMM behavior. A strategy to overcome this problem is known to be the application of an external magnetic field that would break degeneracy among *M*_J_ energetic levels and provoke tunneling conditions to be lost, or at least partly. Therefore, a static field of 1000 Oe was applied to try to suppress the QTM relaxation process. We opt for an external 1000 Oe arbitrary field assuming that it does not necessarily need to match with the optimal applied field for each system but with the aim of comparing all of the results measured under the same experimental conditions. Only compound **10**_**Yb**_ showed signal dependency among frequency. This was an expected feature, since results obtained from Magellan software^37^ demonstrated that the anisotropic axis in a pentanuclear node in compound **6**_**Dy**_ lies perpendicular. The Dy1 anisotropic axis crosses perpendicularly to the Dy2 ion anisotropy axis, an undesired effect since it could counteract the anisotropy effect of the ion. Taking into account that the oblate ion anisotropy axes lie perpendicular toward the ion electronic cloud distribution, we believe that in this particular system, prolate-type ions (such as Er^3+^ and Yb^3+^) are more suitable to design materials showing SMM behavior. This is the case of compounds with prolate-type ions with well-defined axiality, in which the electron distribution is parallel to the anisotropy axis. In the present case, the highest negative net charge among ligand donor atoms comes from the phenoxo bridge giving rise to the shortest bond distances. These bonds are parallelly lined up to the anisotropy axis of prolate ions enhancing ion anisotropy and contributing to a possible SMM behavior (Supporting Information, Figure S9). Many examples of Yb^3+^ SMMs are reported in the literature composed of discrete molecules;^9,38−42^ nonetheless, as far as we know, very few examples of polynuclear Yb^3+^ compounds can be found in the literature, and in most cases, slow relaxation of magnetization is only visible under an applied constant field.^38,43^ In these reported examples, the Yb^3+^ relaxation process occurs preferably by, Raman, direct, and quantum tunneling rather than most usual Orbach processes. Even more surprising, there are no high nuclearity complexes involving Yb^3+^ ions with single-molecule magnet (SMM) behavior that have been covered to date since only discrete molecules or monodimensional coordination polymers have been reported so far.^27,44^ In this sense, compound **10**_**Yb**_ is the first porous three-dimensional metal–organic framework exhibiting SMM behavior in the presence of an external magnetic field.

The relaxation of the magnetization can occur through diverse mechanisms. Purely Orbach type, which follows the Arrhenius law and gives a value of the effective energy barrier directly, although it scarcely happens alone. In most common cases, the relaxation of the magnetization takes place through the combination of several paths, which can be summarized by fitting parameters referred to in eq 1.
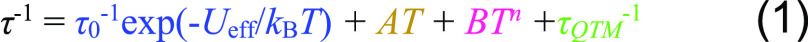
1

The first parameter stands for the Orbach relaxation process, the second parameter (AT) for the direct relaxation path, the third (BT^*n*^) for the Raman relaxation, and τ_QTM_^–1^ makes reference to the quantum tunneling of the magnetization relaxation pathway.

**Figure 3:**
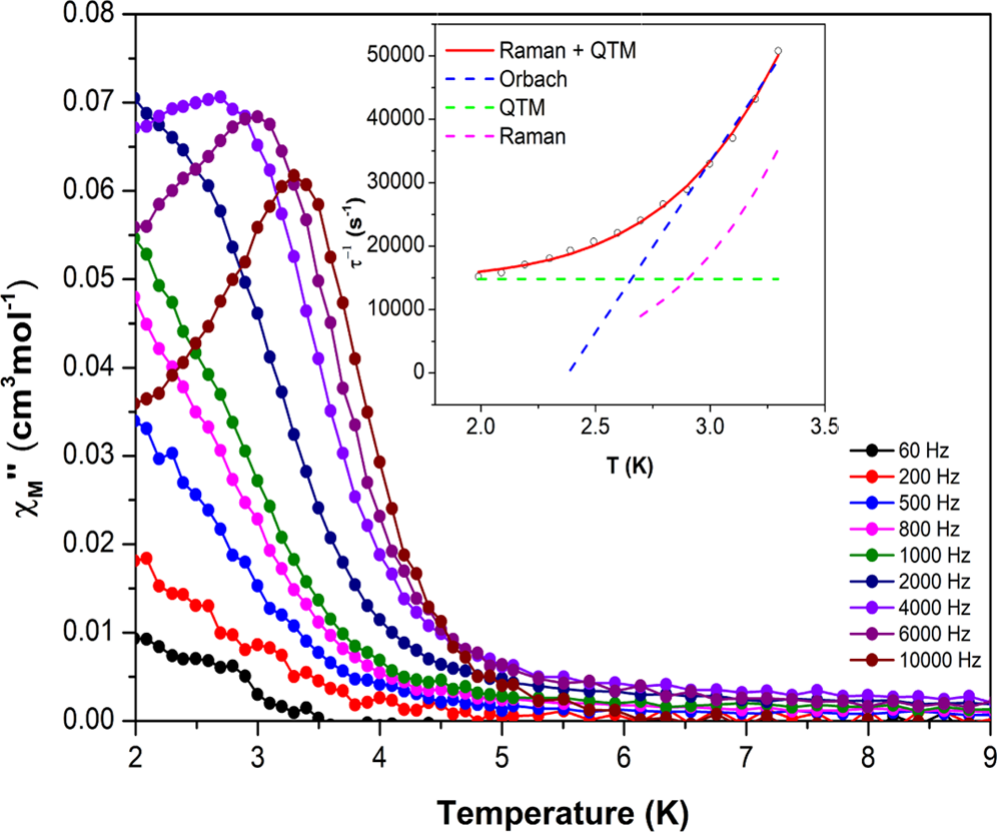
Temperature dependence of out-of-phase components of the ac susceptibility in a dc applied field of 1000 Oe for 10_Yb_. Inset: Arrhenius plot. The black line accounts for the best fit considering Orbach relaxation and the red line corresponds to Raman plus QTM relaxation.

Under an external 1000 Oe field, compound **10**_**Yb**_ reveals slow magnetic relaxation. As previously stated, relaxation in Yb^3+^-based SMMs rarely happens through Orbach processes; therefore, the best fit was obtained taking into consideration Raman and QTM relaxation processes simultaneously (eq 2). These results come in line with the semicircular nature of the Cole–Cole plots and α values (0.28–0.23) and different temperatures, which ward off from 0 values, suggesting that a combination of multiple relaxation processes are taking place. The fitting data were in agreement with the experimental data, as depicted in Figure 3 (inset), affording τ_QTM_ = 6.75 × 10^–5^ s, *B* = 0.56 s^–1^·K^–*n*^, and *n* = 6.802 (Figure 3).
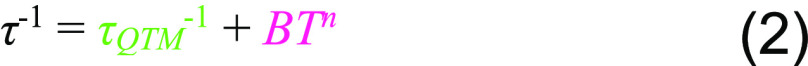
2

However, in view of the residual unquenched QTM occurring in pure samples, we also explored the magnetic dilution strategy with Y^3+^. We tried to isolate paramagnetic centers in a diamagnetic matrix to avoid weak exchange interactions among lanthanide atoms, which could negatively contribute to and favor the single-ion effect. To perform magnetic dilution, we selected a 30:1 Y^3+^ to Ln^3+^ dilution ratio with the aim of isolating a paramagnetic center in each pentanuclear node. The diluted samples were prepared by cocrystallization of the diamagnetic counterpart along with the paramagnetic ion (see Figure S4 for more details on the characterization of doped samples). Following the aforementioned procedure, compounds **12**_**Y-Dy**_, **13**_**Y-Er**_, and **14**_**Y-Yb**_ have been prepared. To explore the slow magnetic relaxation in the diluted samples, magnetic *ac* susceptibilities were measured in the 60–10,000 Hz frequency range. As for pure counterparts, none of the diluted compounds **12**–**14** show frequency-dependent signals without the presence of an external magnetic field (Figures S15, 4, and 5); so, the measurements were repeated by applying a magnetic field of 1000 Oe. In the case of compound **12**_**Y-Dy**_, no maxima can be found in out-of-phase molar magnetic susceptibility, and the frequency-dependent χ_M_″ peaks seem to appear below 2 K, out from the detection limit of the equipment. Fortunately, a pair of maxima and a nice set of maxima in χ_M_″ curves are present for compounds **13**_**Y-Er**_ and **14**_**Y-Yb**_, respectively. These results reinforce our first hypothesis being prolate-type ions more suitable to show single-molecule behavior in this particular system.

**Figure 4:**
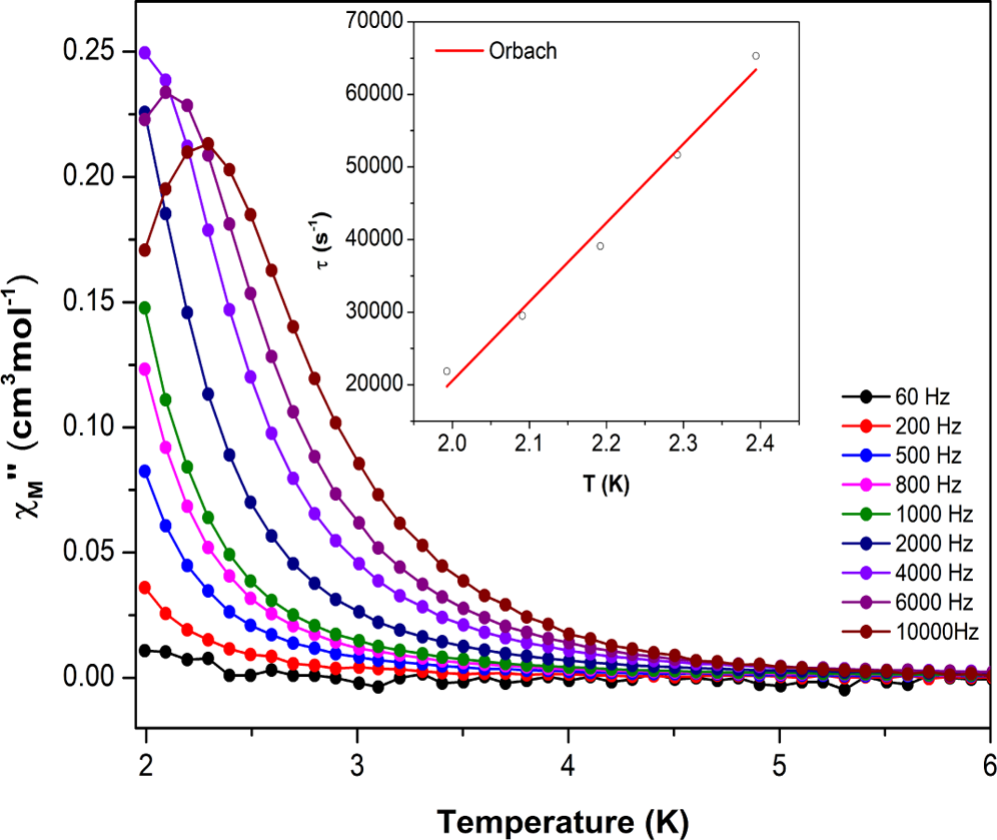
Temperature dependence of out-of-phase components of the ac susceptibility in a dc applied field of 1000 Oe for compound 13_Y-Er_. Inset: Arrhenius plot. The black line accounts for the best fit considering Orbach relaxation.

**Figure 5:**
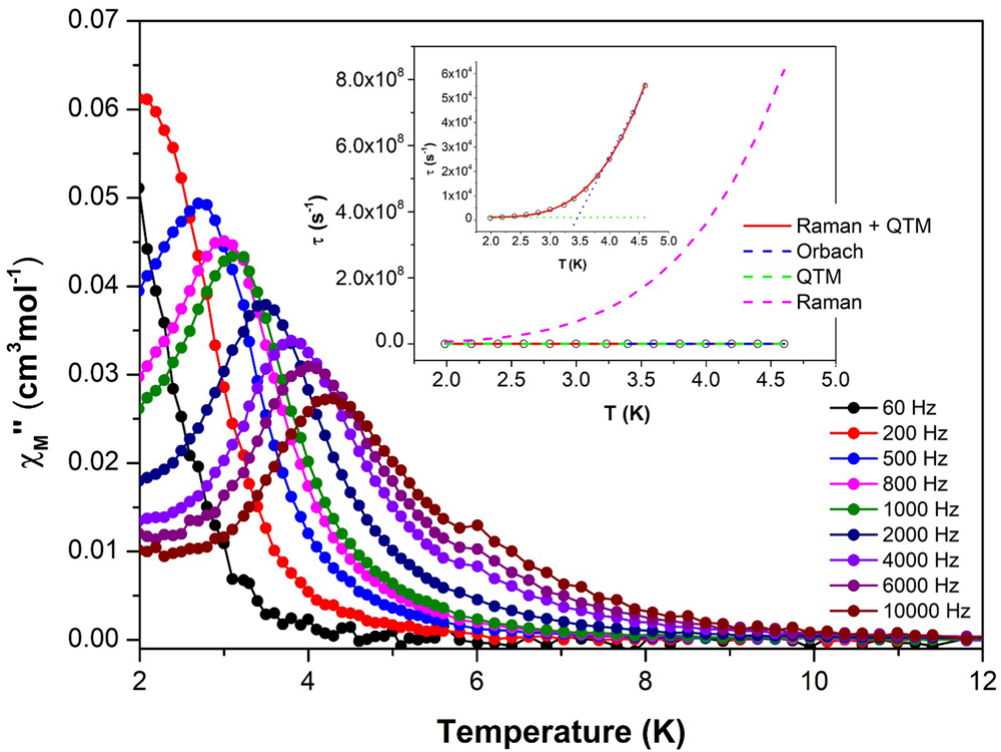
Temperature dependence of out-of-phase components of the ac susceptibility in a dc applied field of 1000 Oe for compound 14_Y-Yb_. Inset: Arrhenius plot. The black line accounts for Orbach fitting and the red line corresponds to the best fit obtained by combining Raman plus QTM relaxation processes.

Despite the fact that compound 12_Y-Dy_ did not show a maximum, the energy barrier (*U*_eff_) and relaxation time (τ_0_) can be estimated if we assume that a single relaxation process is contributing to the ion relaxation. According to the Debye model, applying eq 3, a rough estimation of *U*_eff_ and τ_0_ values can be obtained, yielding *U*_eff_ values of 17.43 K and a relaxation time (τ_0_) of 3.34 × 10^–8^ s^–1^ (Figure S16).
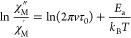
3

In compounds **13**_**Y-Er**_ and **14**_**Y-Yb**_, even after magnetic dilution, a remaining non-negligible fast tunneling relaxation is observed (Figures 4 and 5). However, it seems that is mostly suppressed since better-defined maxima and a shift to higher temperature are obtained. We must notice that the magnetic dilution performed for Er^3+^ in compound **13**_**Y-Er**_ allowed showing SMM behavior. In this compound, the maximum was only clearly visible at 6000 and 10,000 Hz frequencies. The best fitting of the data was achieved with eq 4, which accounts for the Orbach relaxation process, yielding *U*_eff_ values of 13.09 K and a relaxation time (τ_0_) of 6.46 × 10^– 8^ s^–1^.
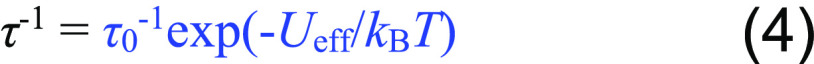
4

In the case of compound **14**_**Y-Yb**_, the relaxation times present a curvature pathway, and as for compound **10**_**Yb**_, the best fitting has been obtained taking Raman and QTM relaxation processes (eq 2) into consideration simultaneously, which gives rise to the following parameters: τ_QTM_ = 2.15 × 10^–2^ s, *B* = 7.2 s^–1^·K^–*n*^, and *n* = 5.87.

### Photoluminescence Properties

Lanthanide-centered emission, characterized by narrow signals in the UV–visible and near-infrared regions, is of great interest given its large applicability in many different areas moving from bioimaging to photovoltaics.^45^ Motivated by these possible applications, in this work, photoluminescence properties have been studied for pure Eu^3+^ (**3**_**Eu**_) and Tb^3+^ (**5**_**Tb**_) compounds as well as for mixtures of lanthanide elements in the Y^3+^-based matrix, using polycrystalline samples in all cases. In particular, our interest was focused on networks bearing Y^3+^ or Gd^3+^, Tb^3+^, and Eu^3+^ in view of their potential application in optical thermometry. Accordingly, three additional heterometallic compounds with Y^3+^ or Gd^3+^ and Tb^3+^/Eu^3+^ mixed lanthanide ions were prepared with the following doping proportions Y^3+^/Tb^3+^/Eu^3+^ 50:45:5 and 50:40:10%, rendering compounds **15**_**Y-Tb-Eu5%**_ and **16**_**Y-Tb-Eu10%**_, respectively, and Gd^3+^/Tb^3+^/Eu^3+^ 50:40:10%, compound **17**_**Gd-Tb-Eu10%**_. With the last two samples, thermometry studies were carried out.

**Figure 6:**
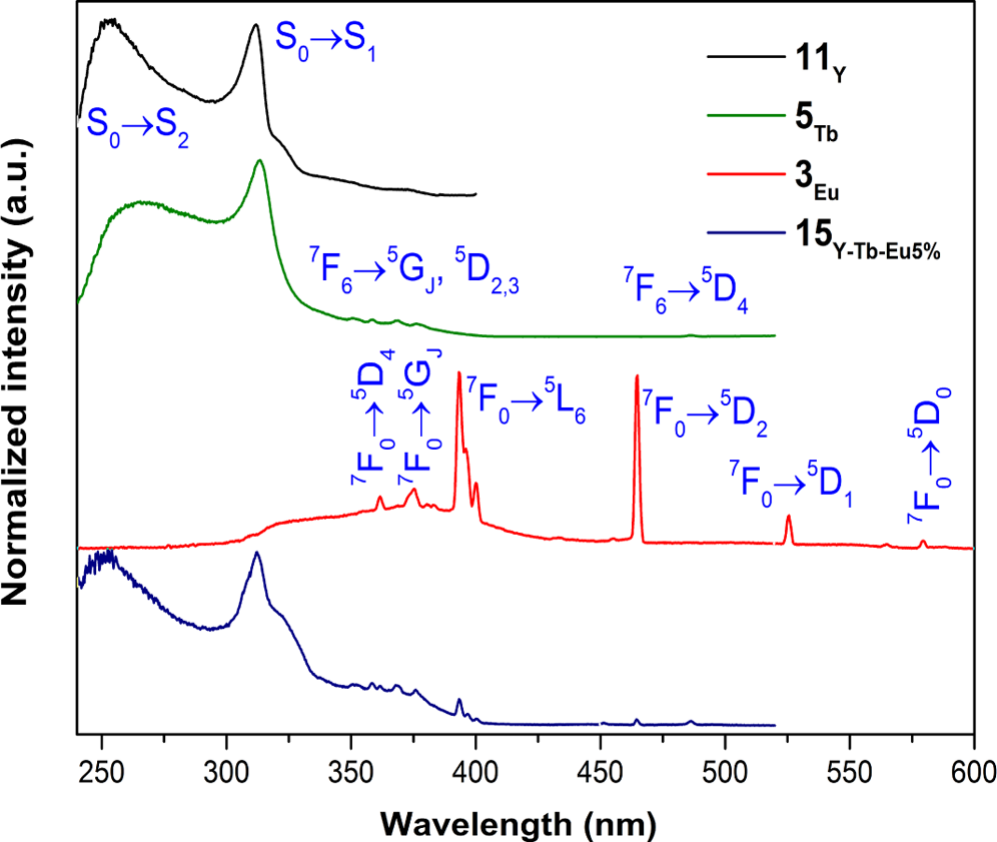
12 K excitation spectra of 11_Y_ (black; λ_Exc._ = 410 nm), 5_Tb_ (green; λ_Exc._ = 544 nm), 3_Eu_ (red; λ_Exc._ = 620 nm), and 15_Y-Tb-Eu5%_ (blue; λ_Exc._ = 614 nm).

The excitation spectra of compounds **11**_**Y**_, **5**_**Tb**_, **3**_**Eu**_, and **15**_**Y-Tb-Eu5%**_ recorded at 12 K are presented in Figure 6. The excitation spectrum of **11**_**Y**_, monitoring the ligand emission at 410 nm, consists of main broad UV bands, ranging from 230 to ca. 380 nm and peaking at 255 and 312 nm, attributed to the transitions from the ground to the low-lying excited states (S_0_ → S_2,1_) of the organic ligand.

On its part, the excitation spectrum recorded for compound **3**_**Eu**_ at 12 K detecting the strongest Eu^3+^ emission at 620 nm is dominated by a set of sharp spectral lines ascribed to the Eu^3+^ intra-4f transitions, from the fundamental ^7^F_0_ level to the ^5^D_0–4_, ^5^L_6_, and ^5^G_J_ excited levels. Although ligand excitation bands are almost absent in this spectrum, some residual ligand signal is still noticeable at wavelengths above 300 nm. Contrary to that, the 12 K excitation spectrum of compound **5**_**Tb**_ is completely dominated by the ligand excitation bands, which are similar to the ones observed for compound **11**_**Y**_. This demonstrates an effective energy transfer from the ligand to the Tb^3+^. The typical Tb^3+^ intra-4f transitions from the fundamental ^7^F_6_ level to the ^5^D_4-2_ and ^5^G_J_ excited levels appear with residual intensities. The excitation spectrum of compound **15**_**Y-Tb-Eu5%**_, monitoring the Eu^3+^ emission at 614 nm, is also dominated by the ligand excitation broad bands with a profile resembling the one of compound **11**_**Y**_, with the maxima peaks slightly shifted to 250 nm and 325 nm, respectively. The most intense Eu^3+^ excitation lines are also present in this spectrum, even if with relatively low intensities. In addition, the first excitation transition of Tb^3+^, ^7^F_6_ → ^5^D_4_ at 485 nm, resulting from the Tb^3+^-to-Eu^3+^ energy transfer process is also identifiable.

**Figure 7:**
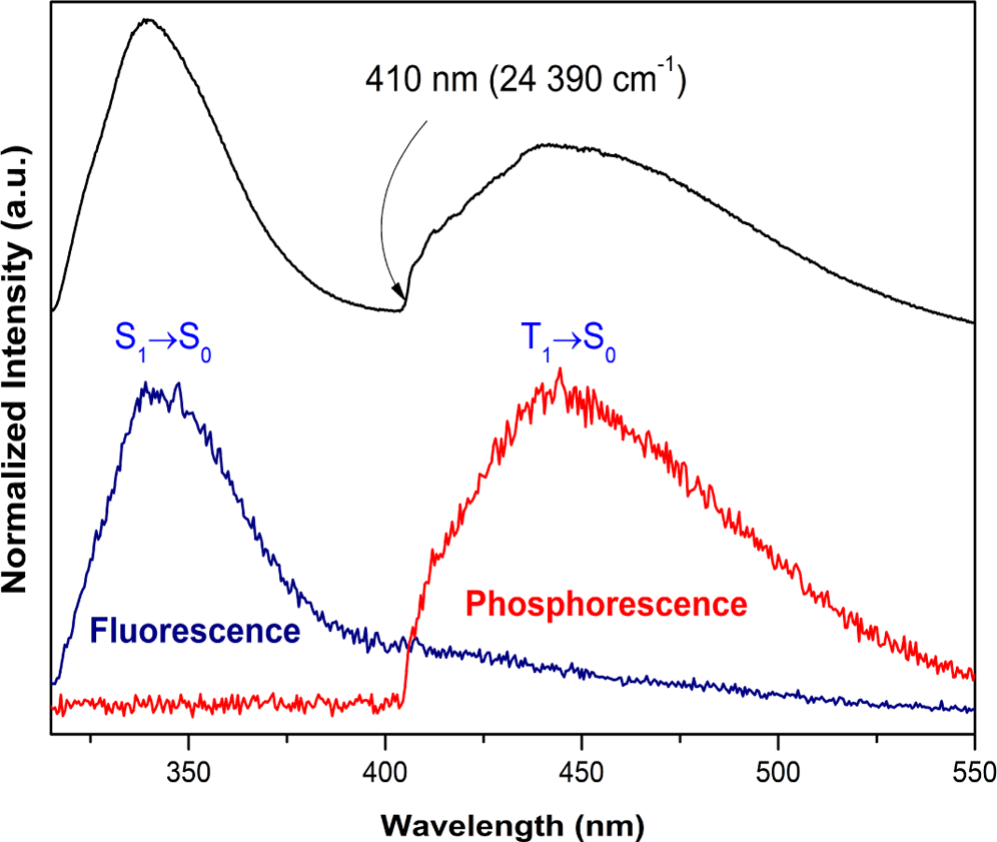
Stationary emission spectra of **11**_**Y**_ (black line) and corresponding time-resolved emission spectra showing the separation of the fluorescence (blue line; initial delay of 0.01 ms and integration time of 0.1 ms) and the phosphorescence (red line; initial delay of 0.1 ms and integration time of 10 ms) recorded at 12 K under 310 nm excitation. The time-resolved spectra were not corrected for the detection and optical spectral response of the spectrofluorometer.

To get deeper insights into the distinct photoluminescence performance of the Tb^3+^ and Eu^3+^ compounds, we have determined the triplet zero-phonon energy of the ligand. For this, stationary-state and time-resolved emission spectra of **11**_**Y**_ have been recorded at 12 K under 310 nm excitation light (Figure 7). As observed, the stationary state emission spectrum displays two broad bands, from 315 to ca. 550 nm, attributed to the S_1_ → S_0_ ligand fluorescence (peaking at 340 nm) and T_1_ → S_0_ ligand phosphorescence (peaking at 450 nm). This assignment is supported by the time-resolved emission spectra, which allowed isolating the fluorescence and phosphorescence emissions using faster and slower detection conditions, respectively. The zero-phonon energy level of the ligand phosphorescence, related to the energy of the emitting triplet states, is estimated at 410 nm (24,390 cm^–1^). This level is relatively close to Tb^3+^ first excited state (^5^D_4_, 585 nm/20,619 cm^–1^) and far from the energy of the lowest-lying excited level of Eu^3+^ (^5^D_0_, 580 nm/17,241 cm^–1^), explaining why the energy transfer is more efficient to the former lanthanide ion according to Latva’s law.^46^

**Figure 8:**
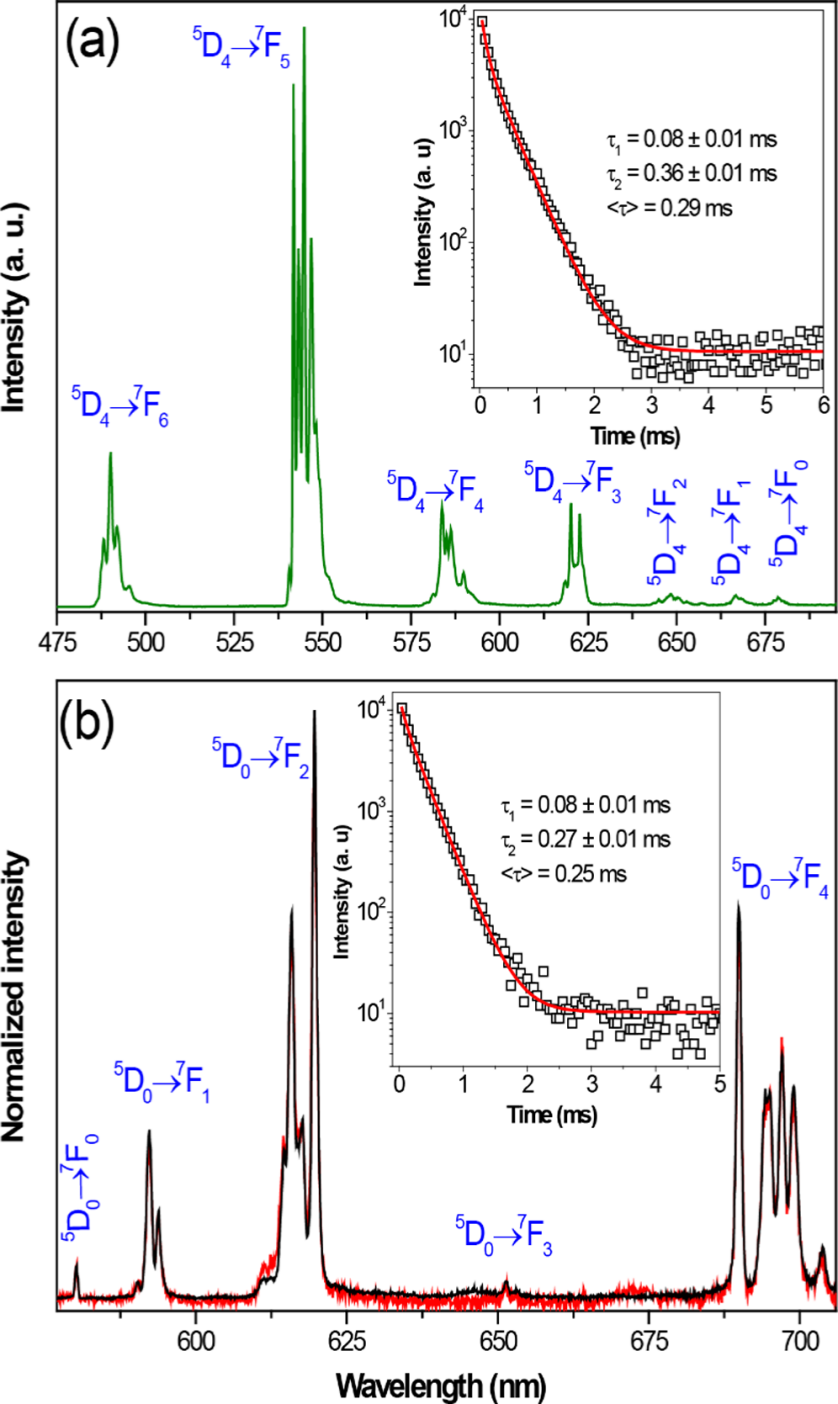
12 K emission spectra of (a) **5**_**Tb**_ (green line) excited at 315 nm and of (b) **3**_**Eu**_ with the excitation fixed at 335 nm (red line) and 393.5 nm (black line). The inserts show the corresponding (a) ^5^D_4_ and (b) ^5^D_0_ decay curves monitoring the emissions at 544.5 and 616 nm with the excitations selected at 377 nm and 393.5 nm, respectively, for **5**_**Tb**_ and **3**_**Eu**_; the solid red lines are the best fits using second-order decay functions, *y* = *y*_0_ + *A*_1_·exp(−*x*/τ_1_) + *A*_2_·exp(−*x*/τ_2_) (*r*^2^ > 0.999). The average lifetimes were calculated according to the formula <τ> = (*A*_1_τ_1_^2^ + *A*_2_τ_2_^2^)/(*A*_1_τ_1_ + *A*_2_τ_2_).

The emission spectrum of **5**_**Tb**_ recorded at 12 K and excited at 315 nm (Figure 8a) presents the typical narrow lines attributed to the Tb^3+ 5^D_4_ → ^7^F_6-0_. The emission spectra of **3**_**Eu**_ recorded at 12 K with the excitation selected at 393.5 nm (^5^L_6_ excited level) and at 335 nm (ligand band) are shown in Figure 8. The spectra exhibit the characteristic sharp intra-4f emission lines of Eu^3+^ attributed to the ^5^D_0_ → ^7^F_0–4_ transitions. In particular, in the ^5^D_0_ → ^7^F_2_ transition region, at least six Stark components can be observed, four main lines and two smaller lines in the low-energy part. The intensity of the two low-energy Stark components clearly increases with the 335 nm excitation. This unequivocally proves the presence of the two independent Eu^3+^ sites as described in the structural section. The dominance of the ^5^D_0_ → ^7^F_2_ transitions over the ^5^D_0_ → ^7^F_1_ transition is typical of Eu^3+^ environments without inversion centers, in line with that previously described in the structural section. In addition, the ^5^D_0_ Eu^3+^ decay curve, recorded at 12 K while monitoring the strongest emission at 616 nm under direct excitation at 393.5 nm (insert of Figure 8b), is only properly fitted by a second-order exponential function, yielding two lifetimes of 0.08 ± 0.01 and 0.27 ± 0.01 ms, and with an averaged lifetime of 0.25 ms. This is again in accordance with the presence of two Eu^3+^ sites in the **3**_**Eu**_ structure. An identical conclusion is obtained for **5**_**Tb**_, for which the corresponding decay curve recorded at 12 K for the emission of the ^5^D_4_ state also yields two lifetimes of 0.08 ± 0.01 and 0.36 ± 0.01 ms, resulting in an averaged lifetime of 0.29 ms.

Temperature-induced changes on the emission spectrum motivated us to studying the capacity of the mixed compounds for luminescent thermometry. For this purpose, we carefully selected compounds **16**_**Y-Tb-Eu10%**_ and **17**_**Gd-Tb-Eu10%**_ because the metals’ mixing proportion is equal among them. Y^3+^ and Gd^3+^ complexes were selected since the former allows Ln–MOF doping within an optically inert Ln matrix and the latter displays high energy of the first excited state, which prevents participation in the studied electron transfer mechanism.^47,48^ Following this strategy, in compounds **16**_**Y-Tb-Eu10%**_ and **17**_**Gd-Tb-Eu10%**_, Ln centers are prompted to be adequately distributed within the net avoiding nonradiative energy-transfer mechanisms derived by intermetallic energy-transfer processes, which could overall reduce luminescence efficiency.^49^

**Figure 9:**
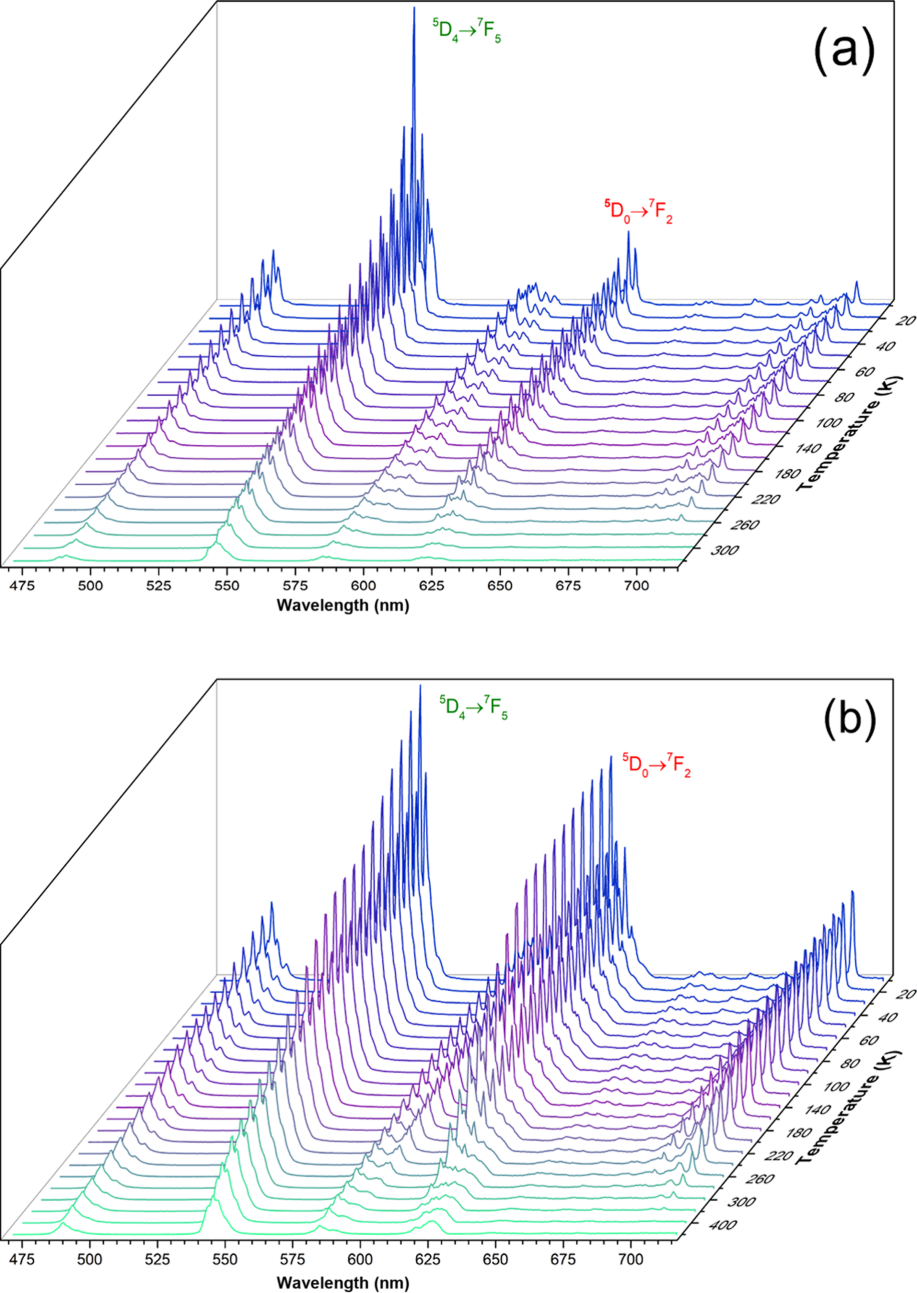
Emission spectra of the 12–320 K range with the excitation selected at 312 nm (a) and emission spectra of **17**_**Gd-Tb-Eu10%**_ in the same range and fixing the excitation at the same wavelength (b).

*I*_Tb_ and *I*_Eu_ were determined by integrating the emission spectra in the ranges of 538–552 and 609–619 nm for **16**_**Y-Tb-Eu10%**_ and in the range of 536–556 and 610–618 nm for **17**_**Gd-Tb-Eu10%**_. Figure 9 presents the temperature-dependent emission spectra of the compounds **16**_**Y-Tb-Eu10%**_ and **17**_**Gd-Tb-Eu10%**_ in the 12–320 K range. As expected, the emission spectra highly resemble to what was obtained for analogous compound **15**_**Y-Tb-Eu5%**_.

**Figure 10:**
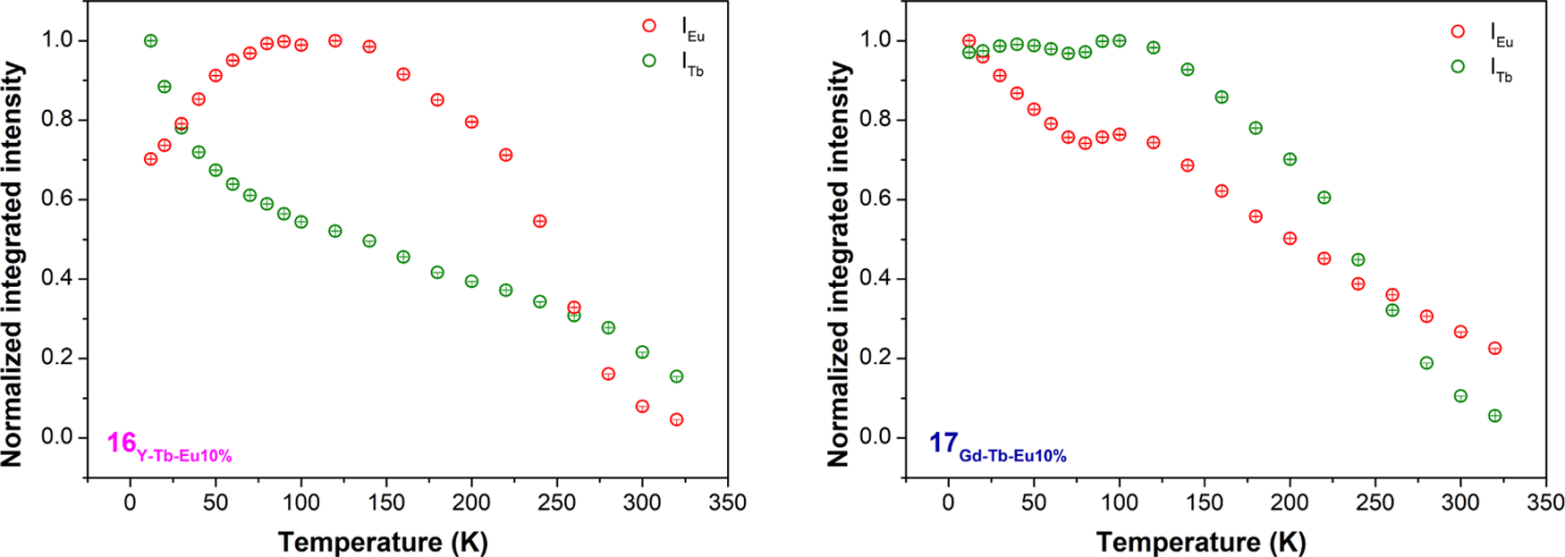
Temperature dependence of *I*_Tb_ (green) and *I*_Eu_ (red) in the 12–320 K range of **16**_**Y-Tb-Eu10%**_ (left) and of **17**_**Gd-Tb-Eu10%**_ (right).

Afterward, the temperature dependence of the integrated intensity of the emissions was calculated and is depicted in Figure 10. Based on the integrated areas of Tb^3+ 5^D_4_ → ^7^F_5_ (*I*_Tb_) and Eu^3+ 5^D_0_ → ^7^F_2_ (*I*_Eu_) emissions, a thermometric parameter may be defined, Δ = *I*_Tb_/*I*_Eu_, allowing to convert emission intensities into absolute temperature values.^21^ An instrumental error of 0.1% was taken to estimate the standard deviation of each experimental data.^50^ The emission of Tb^3+^ decreased by 93 and 94% from 12 to 340 K, and the Eu^3+^ emissions decreased by 65 and 77% for **16**_**Y-Tb-Eu10%**_ and **17**_**Gd-Tb-Eu10%**_ compounds, respectively.

**Figure 11:**
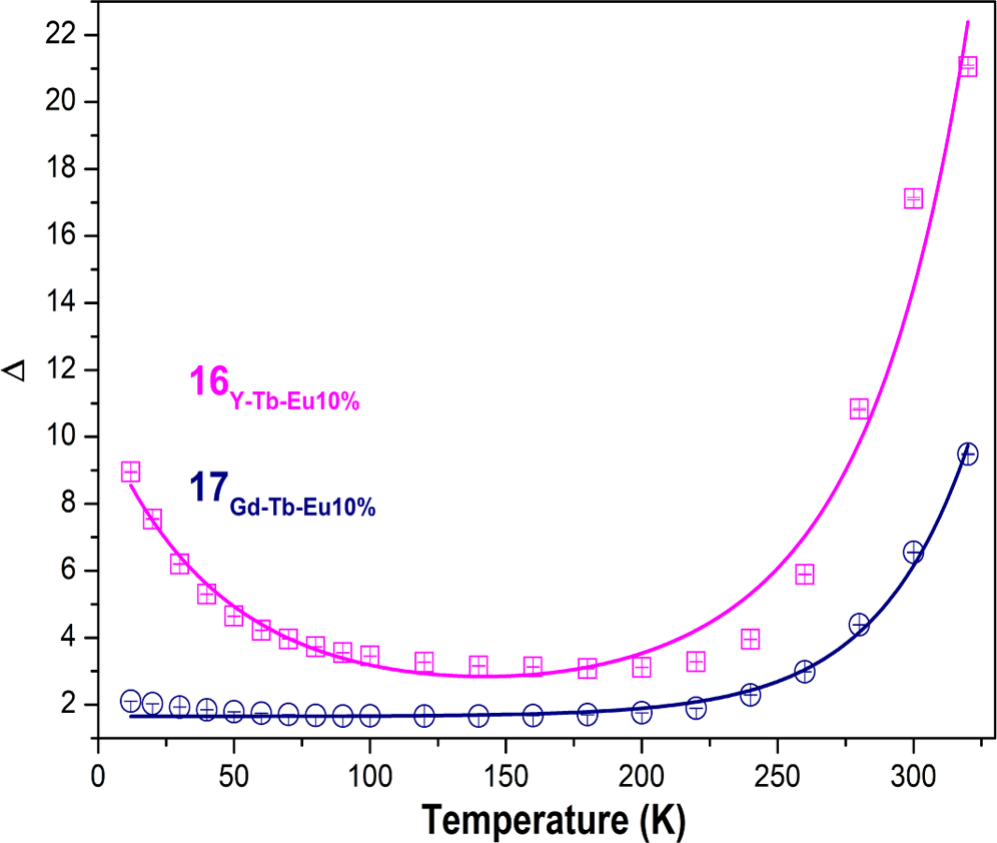
Variation of the ratiometric intensity parameter Δ as a function of temperature in the 12–320 K range for **16**_**Y-Tb-Eu10%**_ in the left side and for **17**_**Gd-Tb-Eu10%**_ in the right side. The solid lines results from the fits considering the following empirical exponential functions: Δ(*T*) = exp(*a* + *bT* + *cT*^2^) (*r*^2^ = 0.96) and Δ(*T*) = Δ_0_ +*A*(*R*_0_*T*) (*r*^2^ = 0.99) for **16**_**Y-Tb-Eu10%**_ and **17**_**Gd-Tb-Eu10%**_, respectively.

**Figure 12:**
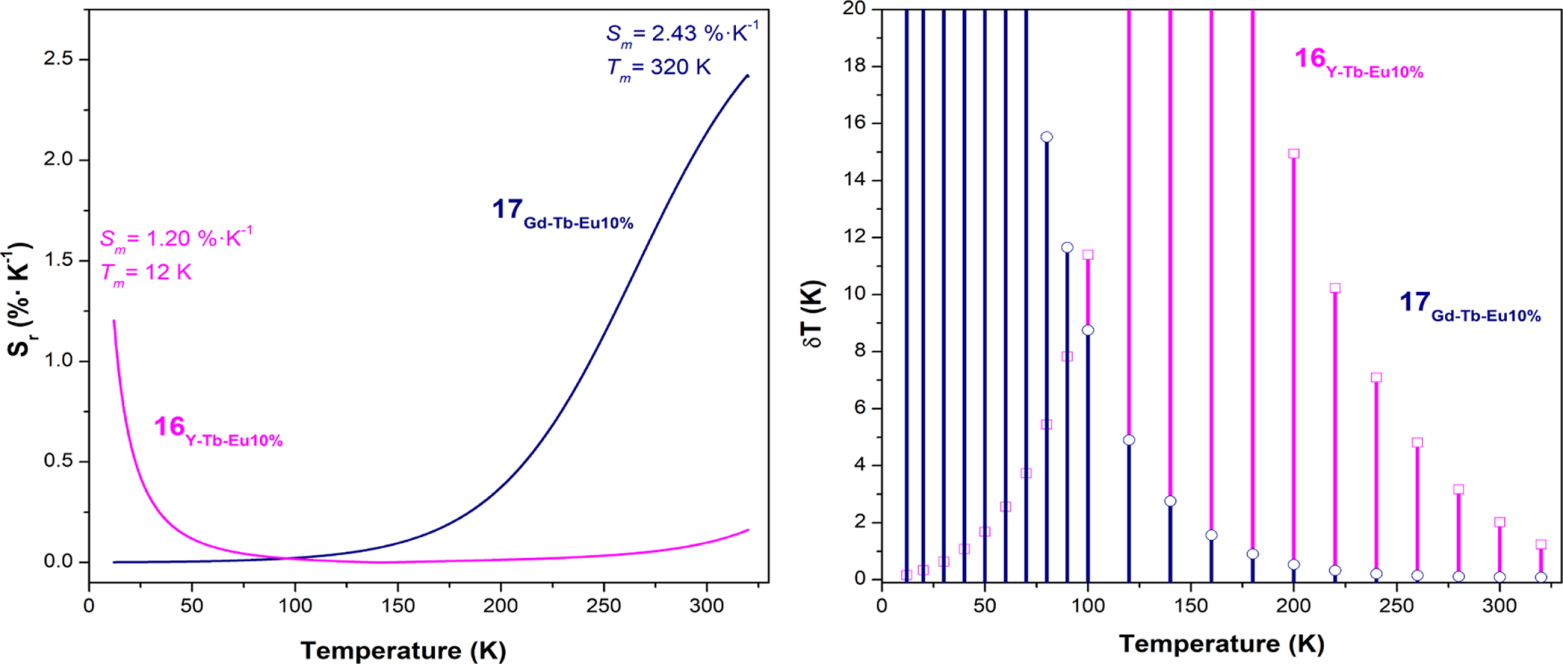
Temperature sensitivity (*S*_r_) in the 12–320 K range for **16**_**Y-Tb-Eu10%**_ and for **17**_**Gd-Tb-Eu10%**_ (left) and the corresponding temperature uncertainty () (right). For clarity, only uncertainty values below 20 K are shown.

The temperature dependence of the thermometric parameter Δ in the range of 12–320 K and the corresponding relative sensitivities, defined as *S*_r_ = |∂Δ|/∂*T* in the same temperature range for compounds **16**_**Y-Tb-Eu10%**_ and **17**_**Gd-Tb-Eu10%**_, are shown in Figures 11 and 12, respectively.

Compound **16** displays two distinct regimens in the sensitivity, most probably originating from the two crystallographic phases identified by PXRD (see Figure S5), whereas for compound **17**, a single temperature-sensing regime can be observed. For **16**_**Y-Tb-Eu10%**_, the maximal relative sensitivity is obtained at *T*_m_ 12 K with *S*_m_ 1.20% K^–1^; the above-mentioned temperature shows a tendency of decreasing relative sensitivity. This behavior is followed until 150 K; afterward, relative sensitivity increases up to 320 K. In the case of compound **17**_**Gd-Tb-Eu10%**_, the maximum relative sensitivity is obtained at *T*_m_ 320 K, yielding *S*_m_ 2.43% K^–1^. These values come in line with the compounds that have been reported so far in the bibliography.^50^ The temperature uncertainty, the minimum temperature change that can be ascertained in a given measurement, is defined as δ*T* = 1/*Sr* (δΔ)/Δ), where δΔ/Δ is the relative error in the determination of the thermometric parameter.^50^ The minimum temperature uncertainties of both compounds (see Figure 12) follow exactly the corresponding maximum sensitivities, 0.08 K at 320 K for **17**_**Gd-Tb-Eu10%**_ and 0.16 K at 12 K for **16**_**Y-Tb-Eu10%**_.

### Adsorption Capacity

With the aim of getting preliminary data on the porosity of the material, the pore size distribution, accessible surface area, and pore volume of **6**_**Dy**_, a Monte Carlo code developed by Herdes and Sarkisov was used.^51,52^ In the light of the results, the calculations (Figure S29) show that coordinated DMF molecules are exposed to voids and their removal generates more accessible pores displaying 3D pores in the range of a 4.89–6.35 Å diameter, exhibiting a surface area of 713.2 m^2^/g and a pore volume of 0.319 cm^3^/g and a porosity of 50.9%.

The porous structure of **6**_**Dy**_ led us to assess its experimental gas adsorption behavior. The accessibility of gaseous probe molecules into the porous framework of **6**_**Dy**_ was first evaluated by recording adsorption isotherms of N_2_ at 77 K. Regretfully, probably due to the narrow pore size of the compound, the study of porosity by means of this gas at 77 K revealed no adsorption capacity. Even though, CO_2_ molecules could diffuse through the porous, as the latter molecule has a smaller kinetic radius compared to the former and comparatively, CO_2_ establishes stronger interactions with the amino group of the ligand. In general, it is known that MOFs with polar (−OH, −N=N–, −NH_2_, and −N=C(R)−) pores show higher CO_2_ adsorption than nonpolar MOFs.^53^ Therefore, CO_2_ adsorption isotherms were recorded at 273 K and 298 K in compound **6**_**Dy**_.

**Figure 13:**
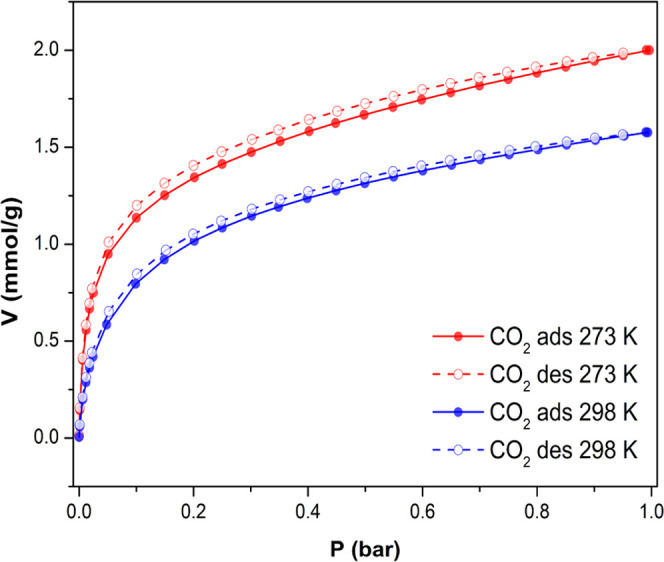
CO_2_ adsorption isotherms at 273 K (red) and 298 K (blue) for **6**_**Dy**_.

Regarding CO_2_ adsorption capacity reaching 1 bar (Figure 13), compound **6**_**Dy**_ loads 2.1 mmol/g at 273 K and 1.6 mmol/g at 298 K. These obtained values can be considered moderate values when compared with those achieved by referential MOFs.^54^ Particularly, obtained adsorption both at 273 K and 298 K comes very good in line with those observed in TMOF-1, which exhibited a CO_2_ uptake of 2.2 mmol/ g (273 K) and 2.2 mmol/g (298 K).^55^ Contrarily, the isosteric heats of CO_2_ adsorption of **6**_**Dy**_ (Figure S28) can be considered as relatively high (see below). In this sense, despite the fact that the low uptake capacity of **6**_**Dy**_ rules out its application in CO_2_ storage, its high adsorption heats makes this material more suitable for separation and purification technologies of this greenhouse gas.^56−59^

At near zero coverage, the *Q*_st_ of CO_2_ in compound **6**_**Dy**_ is 41.4 kJ/mol. This value for CO_2_ is comparable to other previous works,^24^ as well as for some reputed MOFs such as Ni-MOF-74 (42 kJ/mol),^60^ Zn_2_(ox)(atz)_2_ (41 kJ/mol),^61^ and Pd(μ-F-pymo-N^1^,N^3^)_2_ (40 kJ/mol).^62^ Meanwhile, the *Q*_st_ values show a staggering decrease with increasing loading of adsorbate molecules in the MOF, which is related to a gradual decrease in the availability of the best-performing adsorption sites. Precisely, the isosteric heat of the adsorption profile shows three main steps, and when considering *Q*_st_ vs CO_2_ loading per cluster (Figure S27), it could be ascribed to the successive occupation of the three coordinatively unsaturated sites (*cus*) available after the removal of the coordinating solvent molecules during the activation of the MOF (Figure S28).

In comparison to other MOFs containing open metal sites (in which values around 30–60 kJ/mol are obtained),^63^**6**_**Dy**_ exhibits relatively high isosteric heat (41.4 kJ/mol), which also suggests a direct interaction between CO_2_ and *cus*. These coordinatively unsaturated metal sites are available for adsorbate interaction only after carrying the solvent-exchange procedure. As described in the Supporting Information, (Figure S6) the solvent-exchange procedure with MeOH allowed partly/fully replaced coordinated DMF molecules, which by sample activation were removed allowing the structure to contain three *cus* per formula. Generally, as-synthesized MOFs are prone to contain fully coordinated metal ions/clusters with fully completed coordination spheres by bonds formed with solvents and organic ligands. Provided that these bonds can be removed, material activation can provide accessible *cus*, which acts as Lewis acid sites on the surface specifically interacting with gas host molecules. *Cus* is usually the first loading site as it may serve as a charge-dense binding site that strongly interacts with gas molecules. Therefore, it is possible, after the removal of the solvent and subsequent activation of the material, to transform these sites into *cus* that increase adsorbate/surface interactions during the adsorption process as it happens in our particular case.^64^

**Figure 14:**
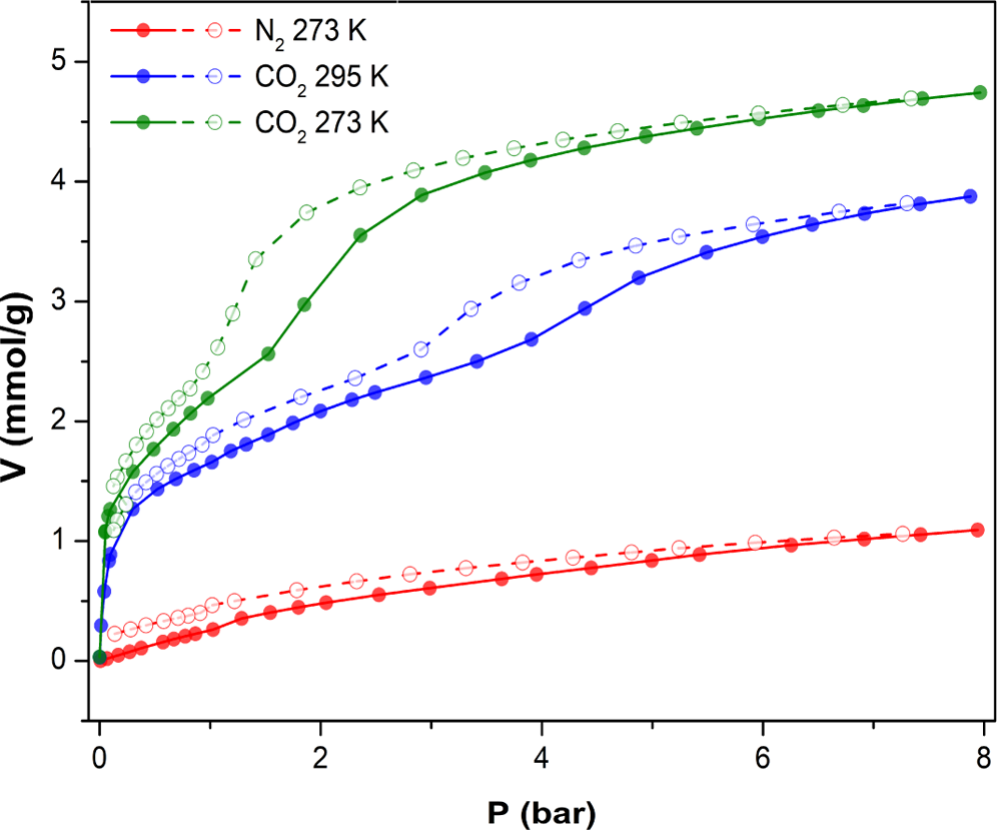
High-pressure adsorption isotherms of N_2_ at 273 K (red) and CO_2_ at 273 K (green) and 298 K (blue), respectively.

We subsequently performed high-pressure adsorption isotherms of N_2_ and CO_2_. As depicted in Figure 14, even at the highest pressure of 8 bar, compound **6**_**Dy**_ shows relatively low affinity toward N_2_ and exhibits an adsorption uptake of 1.10 mmol/g, much lower than that for CO_2_, which are 4.59 mmol/g and 3.84 mmol/g at 273 and 298 K, respectively. Interestingly, the adsorption occurs in two well-distinguishable steps that occur at almost the same loading regardless of the adsorption temperature. Such behavior could be ascribed to the reorganization of CO_2_ at a critical loading to render more room for the next incoming adsorbate molecules.

## Conclusions

We report a family of novel three-dimensional metal–organic frameworks based on 3-amino-4-hydroxybenzoate and Ln^3+^ ions. These coordination networks are isostructural among them and crystallize in the hexagonal *P*6_3_/*m* space group and are formulated as {[Ln_5_L_6_(OH)_3_(DMF)_3_]·5H_2_O}*_n_*. With the aim of exploiting the potential multifunctional character of these Ln–MOFs, their single-molecule magnet behavior, photoluminescent properties, and adsorption capacity have been studied. The magnetic properties of the studied materials (namely **6**_**Dy**_, **8**_**Er**_, and **10**_**Yb**_) exhibit frequency dependence without an external magnetic field due to the effect of quantum tunneling of magnetization. When a 1000 Oe static field was applied to suppress the QTM relaxation process, only compound **10**_**Yb**_ showed signal dependency among frequency, which, as far as we are aware, constitutes the first porous three-dimensional Yb-based MOF exhibiting field-induced SMM behavior. In view of residual unquenched QTM occurring in pure samples, a magnetic dilution strategy was performed (in this case with Y^3+^), yielding compounds **12**_**Y-Dy**_, **13**_**Y-Er**_, and **14**_**Y-Yb**_. The procedure of isolating paramagnetic centers in a diamagnetic matrix was successful in the latter two compounds since the position of the maxima in χ_M_″ was shifted toward higher temperatures. Compounds **13**_**Y-Er**_ and **14**_**Y-Yb**_ present Orbach-(with *U*_eff_ = 13.09 K and τ_0_ = 6.46 × 10^–8^ s^–1^) and QTM plus Raman-(τ_QTM_ = 2.15 × 10^–2^ s, *B* = 7.2 s^–1^·K^–*n*^ and *n* = 5.87) mediated relaxation mechanisms, whereas compound **12**_**Y-Dy**_ shows no maxima in out-of-phase molar magnetic susceptibility. These results may be explained according to the electron density shape of the lanthanide(III) ion since the system benefits prolate-type ions (Er^3+^ and Yb^3+^) rather than oblate ions (Dy^3+^).

On another level, compounds **3**_**Eu**_ and **5**_**Tb**_ present characteristic emissions of the ions, among which a Tb^3+^ ion shows more brilliant and long-lived emission than Eu^3+^ because the ligand-to-lanthanide energy transfer is more efficient for the former owing to the low-energy gap between their excited states. Three additional Y^3+^-or Gd^3+^-and Tb^3+^/Eu^3+^-mixed lanthanide networks were prepared with the following Y^3+^/Tb^3+^/Eu^3+^ doping proportions of 50:45:5% (**15**) and 50:40:10% (**16**) and Gd^3+^/Tb^3+^/Eu^3+^ of 50:40:10% for compound **17** and exploited for potential application in thermometry. Interestingly, compounds **16** and **17** performed as luminescent thermometers showing a maximal relative sensitivity of *S*_m_ 1.202% K^–1^ obtained at *T*_m_ 12 K and *S*_m_ 2.43% K^–1^ at *T*_m_ 320 K, respectively.

This family of compounds possesses porous structures characterized by narrow microchannels along the *c* axis which additionally allowed us to explore the adsorption capacity of the synthesized materials at low-pressure and high-pressure conditions. Although compound **6**_**Dy**_’s adsorption isotherms of N_2_ revealed no adsorption capacity, CO_2_ molecules could diffuse through the pores exhibiting 2.1 mmol/g uptake of CO_2_ physisorbed at 273 K (at STP conditions), and a volume of 1.6 mmol/g at 298 K. In comparison to other MOFs containing open metal sites, **6**_**Dy**_ exhibits relatively high isosteric heat (41.4 kJ/mol) that supports the strong interaction between CO_2_ and the first of the three *cus* centers present in the network. Upon loading the framework with the gas, the interaction progressively decreases showing two more steps in the profile that perfectly match the coordination of CO_2_ with the remaining two *cus* pertaining to the pentanuclear cluster.

## Supporting Information Available

Further details on characterization of materials and magnetic and luminescence and gas adsorption experiments (PDF)
