# Supplementary material for: Multifunctional Lanthanide-Based Metal–Organic Frameworks Derived from 3-Amino-4-hydroxybenzoate: Single-Molecule Magnet Behavior, Luminescent Properties for Thermometry, and CO2 Adsorptive Capacity
Source: Inorg Chem. 2022 Aug 8;61(33):12977–90. doi: 10.1021/acs.inorgchem.2c00544 (PMC9406282; doi:10.1021/acs.inorgchem.2c00544)
Supplement: Supplementary file 1 — ic2c00544_si_001.pdf [file ic2c00544_si_001.pdf]

Supporting Information for:

Multifunctional Lanthanide-based Metal-organic Frameworks derived from 3-amino-4-hydroxybenzoate: Single-molecule magnet behaviour. luminescent properties for thermometry and CO_2 adsorptive capacity

Estitxu Echenique-Errandonea,[a] Ricardo F. Mendes,[b] Flavio Figueira,[b] Duane Choquesillo-Lazarte,[c] Garikoitz Beobide,[d] Javier Cepeda,[a] Duarte Ananias,[b] Antonio Rodríguez-Diéguez,*[e] Filipe A. Almeida Paz*[b] and José M. Seco*[a]

[a] Departamento de Química Aplicada, Facultad de Química, Universidad del País Vasco UPV/EHU, Paseo Manuel Lardizabal, Nº 3, 20018, Donostia-San Sebastián, Spain. E-mail: josemanuel.seco@ehu.eus

[b] Department of Chemistry, CICECO – Aveiro Institute of Materials, University of Aveiro, 3810-193 Aveiro, Portugal. E-mail: filipe.paz@ua.pt

[c] Laboratorio de Estudios Cristalográficos, IACT, CSIC-UGR, Av. Las Palmeras nº4, 18100 Granada, Spain.

[d] BCMaterials, Basque Center for Materials, Applications and Nanostructures, UPV/EHU Science Park, 48940 Leioa, Spain.

[e] Departamento de Química Inorgánica, Facultad de Ciencias, Universidad de Granada, Av. Fuentenueva S/N, 18071 Granada, Spain. E-mail: antonio5@ugr.es

Table of Contents:

Experimental Section 3
1.1. General Instrumentation 3
1.2. Single-crystal structure determination 3
Chemical characterization of compounds 4
1.3. Elemental analysis 4
FT-IR spectroscopy 5
Crystallographic data 6
Selected bond lengths and angles data 7
Powder X-ray diffraction analysis 9
Continuous Shape Measurements 12
Thermal analysis 13
Thermal evolution 15
Additional views of the structure 16
Ac magnetic susceptibility measurements 17
Scanning Electron Microscopy 23
Photoluminescence measurements 27
Adsorption properties 29
References 32

Experimental Section

1.1. General Instrumentation

Elemental analyses (C. H. N) were performed on a Leco CHNS-932 microanalyser. Infrared (IR) spectra (400-4000 cm^{-1}) were recorded on a Nicolet FT-IR 6700 spectrometer in KBr pellets.

Thermogravimetric analysis (TG/DTA) were performed on a TG-Q500 TA Instruments thermal analyser from room temperature to 800 °C under a synthetic air atmosphere (79 % N_2/21 % O_2) at a heating rate of 10 °C min^{-1}.

Scanning electron microscopy (SEM) images were acquired using either a Hitachi S4100 field emission gun tungsten filament instrument working at 25 kV or a high-resolution Hitachi SU-70 working at 4 kV. Samples were prepared by deposition on aluminium sample holders followed by carbon coating using an Emitech K950X carbon evaporator. EDS (energy dispersive X-ray spectroscopy) data and SEM mapping images were recorded using the latter microscope working at 15 kV and using either a Bruker Quantax 400 or an Esprit 1.9 EDS microanalysis system.

Magnetic susceptibility measurements were performed on polycrystalline samples of the complexes with a Quantum Design SQUID MPMS-7T susceptometer at an applied magnetic field of 1000 G. The susceptibility data were corrected for diamagnetism estimated from Pascal's tables.[1] the temperature-independent paramagnetism and magnetisation of the sample holder. The ac measurements were performed on a physical property measurement system quantum design model 6000 magnetometer under a 3.5 G ac field and frequencies ranging from 60 to 10000 Hz.

Photoluminescence Spectroscopy. The emission and excitation spectra were recorded at ambient-temperature and 12 K using a Fluorolog®-3Horiba Scientific (Model FL3-2T) spectroscope. with a modular double grating excitation spectrometer (fitted with a 1200 grooves/mm grating blazed at 330 nm) and a TRIAX 320 single emission monochromator (fitted with a 1200 grooves/mm grating blazed at 500 nm. reciprocal linear density of 2.6 $nm.mm^{-1}$). coupled to a R928 Hamamatsu photomultiplier. using the front face acquisition mode. The excitation source was a 450 W Xe arc lamp. The emission spectra were corrected for detection and optical spectral response of the spectrofluorimeter and the excitation spectra were corrected for the spectral distribution of the lamp intensity using a photodiode reference detector. Time-resolved measurements have been carried out using a 1934D3 phosphorimeter coupled to the Fluorolog®-3. and a Xe-Hg flash lamp (6 μs/pulse half width and 20-30 μs tail) was used as the excitation source. The low temperature measurements (12 K) were performed using a helium-closed cycle cryostat with vacuum system measuring ca. 5×10^{-6} mbar and a Lakeshore 330 auto-tuning temperature controller with a resistance heater.

N_2 (77 and 273 K) and CO_2 (273 and 298 K) physisorption data were measured in a Quantachrome Autosorb-iQ MP. Prior to measurements, all samples were outgassed under vacuum at 150 °C for 6 hours. To estimate CO_2 adsorption enthalpies (Q_{st}), the isotherms were fitted to the modified Clausius−Clapeyron equation.

1.2. Single-crystal structure determination

X-ray data collection of suitable single crystals were done at 100(2) K on a Bruker D8 VENTURE area detector equipped with graphite monochromated Mo−Kα radiation (λ = 0.71073 Å) by applying the ω-scan method. The data reduction was performed with the APEX270 software and corrected for absorption using SADABS.[2] Crystal structures were solved by direct methods using the SIR97 program[3] and refined by full-matrix least-squares on F2 including all reflections using anisotropic displacement parameters by means of the WINGX[4] crystallographic package. All hydrogen atoms were included as fixed contributions riding on attached atoms

with isotropic thermal displacement parameters 1.2 times or 1.5 times those of their parent atoms for the organic ligands. Lattice solvent molecules could not be refined owing to their disordered disposition in the voids of the structures. so the electron density at the voids was subtracted from the reflection data by the SQUEEZE procedure as implemented in PLATON program[5] during the refinement. Moreover. some soft constraints concerning the coordinated DMF molecules had to be employed for the final refinement of the structure of compound $\mathbf{6_{Dy}}$. Details of the structure determination and refinement of compound $\mathbf{6_{Dy}}$ are summarized in Table S2. Crystallographic data for the structures reported in this paper have been deposited with the Cambridge Crystallographic Data Center as supplementary publication. CCDC 2151343.

The X-ray powder diffraction (XRPD) patterns were collected at 25 °C on a Phillips X'PERT powder diffractometer with Cu-Kα radiation (λ = 1.5418 Å) over the range 5 < 2θ < 50° with a step size of 0.02° and an acquisition time of 2.5 s per step. Indexation of the diffraction profiles were made by means of the FULLPROF program (pattern- matching analysis) based on the space group and the cell parameters found by single crystal X-ray diffraction.[6]

Variable-temperature powder X-ray diffraction measurements were conducted on a Bruker D8 Advance diffractometer. using polycrystalline sample of compound $\mathbf{6_{Dy}}$ under ambient atmosphere with heating rate of 5 °C·min^{-1} and measuring a complete diffractogram every 20 °C up to 510 °C. and every 50 °C from 510 °C up to 710 °C

Chemical characterization of compounds

1.3. Elemental analysis

Table S1. Elemental analysis of compounds 1-10.

Compound	Formula	Molecular weight	Calc.	Found.
**1**	$C_{51}H_{64}N_9O_{29}Nd_5$	1988.31	C: 30.80; H: 3.24; N: 6.34; O: 23.34; Nd: 36.27	C: 30.82; H: 3.21; N: 6.35; O: 23.37; Nd: 36.24
**2**	$C_{51}H_{64}N_9O_{29}Sm_5$	2019.11	C: 30.34; H: 3.19; N: 6.24; O: 22.98; Sm: 37.24;	C: 30.33; H: 3.21; N: 6.26; O: 22.97; Sm: 37.25;
**3**	$C_{51}H_{64}N_9O_{29}Eu_5$	2026.91	C: 30.22; H: 3.18; N: 6.22; O: 22.89; Eu: 37.49;	C: 30.23; H: 3.19; N: 6.22; O: 22.91; Eu: 37.49;
**4**	$C_{51}H_{64}N_9O_{29}Gd_5$	2053.36	C: 29.83; H: 3.14; N: 6.14; O: 22.60; Gd: 38.29;	C: 29.82; H: 3.13; N: 6.16; O: 22.60; Gd: 38.30;
**5**	$C_{51}H_{64}N_9O_{29}Tb_5$	2061.74	C: 29.71; H: 3.13; N: 6.11; O: 22.50; Tb: 38.54;	C: 29.72; H: 3.13; N: 6.13; O: 22.54; Tb: 38.55;
**6**	$C_{51}H_{64}N_9O_{29}Dy_5$	2079.61	C: 29.46; H: 3.10; N: 6.06; O: 22.31; Dy: 39.07;	C: 29.48; H: 3.12; N: 6.05; O: 22.33; Dy: 39.07;
**7**	$C_{51}H_{64}N_9O_{29}Ho_5$	2091.76	C: 29.28; H: 3.08; N: 6.03; O: 22.18; Ho: 39.42;	C: 29.29; H: 3.11; N: 6.05; O: 22.20; Ho: 39.43;
**8**	$C_{51}H_{64}N_9O_{29}Er_5$	2103.41	C: 29.12; H: 3.07; N: 5.99; O: 22.06; Er: 39.76;	C: 29.12; H: 3.10; N: 6.00; O: 22.08; Er: 39.82;
**9**	$C_{51}H_{64}N_9O_{29}Tm_5$	2111.78	C: 29.01; H: 3.05; N: 5.97; O: 21.97; Tm: 40.00;	C: 29.03; H: 3.06; N: 5.97; O: 21.97; Tm: 40.02;
**10**	$C_{51}H_{64}N_9O_{29}Yb_5$	2132.31	C: 28.73; H: 3.03; N: 5.91; O: 21.76; Yb: 40.58;	C: 28.74; H: 3.06; N: 5.93; O: 21.76; Yb: 40.56;

FT-IR spectroscopy

FTIR spectra of compound $\mathbf{6_{Dy}}$ display a narrow peak at around 3625 cm^{-1}. attributed to the N-H stretching vibration of amine group. which is practically hided below the intense broad band around 3412 cm^{-1} attributed to O–H bond vibration of the of 3-amino-4-hydroxybenzoate free ligand.

At lower frequency. a set of intense bands are visible between 3207 cm^{-1} and 2921–2830 cm^{-1} which corresponds to aromatic ring's C–H bond vibrations of the ligand The intense vibrations in the 1661–1433 cm^{-1} region are referred to both the asymmetric stretching vibrations of the carboxylate groups and the aromatic C–C and C–N bonds. The symmetric stretching vibrations of the carboxylate groups appear in the lower range of 1381–1281 cm^{-1}. The remaining bands that are found at lower frequency can be attributed to the distortions originated in the aromatic ring and the carboxylate groups of the ligands. The vibration bands of the M–O and M–N bonds are observed below 646cm^{-1}.

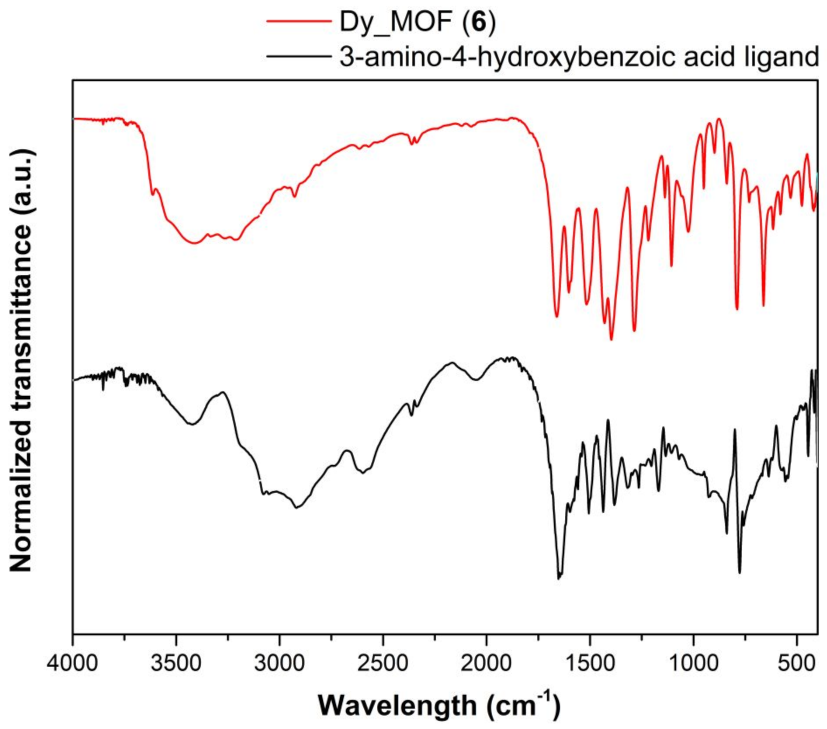

Figure S1. Infrared spectra of the ligand and compound $\mathbf{6_{Dy}}$.

Crystallographic data

Table S2. Crystallographic data and structure refinement details of compound 6.

**Compound**	$\mathbf{6_{Dy}}$
Formula	$C_{51}H_{51}N_9O_{24}Dy_5$
M_r	1986.51
Crystal system	hexagonal
Space group (no.)	*P*63/*m*
a(Å)	15.4579(11)
b(Å)	15.4579(11)
c(Å)	17.0864(13)
α(°)	90
β(°)	90
γ(°)	120
V(Å^3)	3535.8(6)
Z	4
ρ_{calc}g/cm^3	1.866
μ/mm^{-1}	5.293
F(000)	1884.0
Crystal size/mm^3	0.27 × 0.24 × 0.22
Radiation	MoKα (λ = 0.71073)
2θ range for data collection/°	3.866 to 50.038
Index ranges	$-18 \le h \le 17$. $-18 \le k \le 18$. $-20 \le l \le 20$
Reflections collected	23375
Independent reflections	2170 [R_{int} = 0.1243. R_{sigma} = 0.0634]
Data/restraints/parameters	2170/57/160
Goodness-of-fit on F^2	1.016
Final R indexes [I>=2σ (I)]	R_1 = 0.0394. wR_2 = 0.0837
Final R indexes [all data]	R_1 = 0.0666. wR_2 = 0.0944
Largest diff. peak/hole / e Å^{-3}	1.09/-1.31

Selected bond lengths and angles data

Table S3. Table of the s elected bond lengths (Å) and angles (°) for compound 6_{Dy}.

Complex		6_{Dy}
Dy1	Dy1^{1}	3.4984(12)
Dy1	Dy2^{2}	3.9242(6)
Dy1	Dy2	3.9242(6)
Dy1	O1H^{3}	2.366(5)
Dy1	O1H	2.366(5)
Dy1	O1H^{2}	2.366(5)
Dy1	O3	2.513(6)
Dy1	O3^{2}	2.513(6)
Dy1	O3^{3}	2.513(6)
Dy1	N1	2.509(7)
Dy1	N1^{3}	2.510(7)
Dy1	N1^{2}	2.510(7)
Dy2	O1^{4}	2.394(6)
Dy2	O1^{5}	2.394(6)
Dy2	O1H	2.337(7)
Dy2	O2^{4}	2.417(6)
Dy2	O2^{5}	2.417(6)
Dy2	O3	2.371(5)
Dy2	O3^{1}	2.371(5)
Dy2	O4	2.327(11)

^{1}+x,+y,3/2-z; ^{2}1-y,1+x-y,+z; ^{3}+y-x,1-x,+z; ^{4}+y,1-x+y,1/2+z; ^{5}+y,1-x+y,1-z

Dy1	Dy2	Dy11	52.943(18)	O1H2	Dy1	O1H3	71.4(2)
Dy11	Dy1	Dy22	63.527(9)	O1H2	Dy1	O33	74.5(2)
Dy11	Dy1	Dy2	63.528(9)	O1H2	Dy1	O3	133.58(17)
Dy11	O1H	Dy1	95.3(3)	O1H2	Dy1	O32	68.5(2)
Dy2	O1H	Dy1	113.1(2)	O1H2	Dy1	N1	142.4(2)
Dy2	O1H	Dy11	113.1(2)	O1H2	Dy1	N13	88.4(2)
Dy2	O3	Dy1	106.89(19)	O1H2	Dy1	N12	132.4(2)
Dy22	Dy1	Dy2	101.651(11)	O1H3	Dy1	Dy11	42.34(13)
N1	Dy1	Dy11	130.44(18)	O1H3	Dy1	Dy2	69.01(17)
N1	Dy1	Dy22	158.59(18)	O1H3	Dy1	Dy22	101.91(14)
N1	Dy1	Dy2	99.55(17)	O1H3	Dy1	O3	74.5(2)
N1	Dy1	O3	64.6(2)	O1H3	Dy1	O32	133.59(17)
N1	Dy1	O33	68.7(2)	O1H3	Dy1	O33	68.5(2)
N1	Dy1	O32	138.0(2)	O1H3	Dy1	N12	142.4(2)
N12	Dy1	Dy11	130.44(19)	O1H3	Dy1	N1	88.4(2)
N12	Dy1	Dy22	99.55(17)	O1H3	Dy1	N13	132.4(2)
N12	Dy1	Dy2	76.73(17)	O24	Dy2	Dy1	162.03(15)
N12	Dy1	O3	68.7(2)	O24	Dy2	Dy11	112.49(16)
N12	Dy1	O32	64.6(2)	O24	Dy2	O25	79.3(3)

N12	Dy1	O33	138.0(2)	O25	Dy2	Dy1	112.49(16)
N12	Dy1	N1	82.5(3)	O25	Dy2	Dy11	162.03(15)
N13	Dy1	Dy11	130.44(19)	O3	Dy1	Dy11	91.44(12)
N13	Dy1	Dy22	76.73(17)	O3	Dy1	Dy22	136.10(12)
N13	Dy1	Dy2	158.59(18)	O3	Dy1	Dy2	35.32(12)
N13	Dy1	O32	68.7(2)	O3	Dy1	O33	119.937(11)
N13	Dy1	O3	138.0(2)	O3	Dy2	Dy1	37.79(13)
N13	Dy1	O33	64.6(2)	O3	Dy2	Dy11	83.76(14)
N13	Dy1	N1	82.5(3)	O3	Dy2	O14	145.2(2)
N13	Dy1	N12	82.5(3)	O3	Dy2	O15	82.1(2)
O14	Dy2	Dy1	109.19(15)	O3	Dy2	O24	160.2(2)
O14	Dy2	Dy11	76.71(17)	O3	Dy2	O25	88.0(2)
O14	Dy2	O15	77.6(3)	O3	Dy2	O31	99.7(3)
O14	Dy2	O25	101.9(2)	O31	Dy2	Dy11	37.80(13)
O14	Dy2	O24	53.7(2)	O31	Dy2	Dy1	83.76(14)
O15	Dy2	Dy1	76.71(17)	O31	Dy2	O14	82.1(2)
O15	Dy2	Dy11	109.19(15)	O31	Dy2	O15	145.2(2)
O15	Dy2	O25	53.7(2)	O31	Dy2	O24	88.0(2)
O15	Dy2	O24	101.9(2)	O31	Dy2	O25	160.2(2)
O1H	Dy1	Dy11	42.34(13)	O32	Dy1	Dy11	91.44(12)
O1H	Dy1	Dy22	69.01(17)	O32	Dy1	Dy2	97.41(13)
O1H	Dy1	Dy2	33.21(16)	O32	Dy1	Dy22	35.32(12)
O1H	Dy1	O1H3	71.4(2)	O32	Dy1	O3	119.938(12)
O1H	Dy1	O33	133.59(17)	O32	Dy1	O33	119.938(12)
O1H	Dy1	O32	74.5(2)	O33	Dy1	Dy11	91.44(12)
O1H	Dy1	O3	68.5(2)	O33	Dy1	Dy2	136.10(12)
O1H	Dy1	N13	142.4(2)	O33	Dy1	Dy22	97.41(13)
O1H	Dy1	N12	88.4(2)	O4	Dy2	Dy11	111.9(8)
O1H	Dy1	N1	132.4(2)	O4	Dy2	Dy1	112.3(4)
O1H	Dy2	Dy1	33.69(10)	O4	Dy2	O14	132.7(7)
O1H	Dy2	Dy11	33.69(10)	O4	Dy2	O15	133.4(10)
O1H	Dy2	O14	76.4(2)	O4	Dy2	O1H	136.4(5)
O1H	Dy2	O15	76.4(2)	O4	Dy2	O25	82.5(9)
O1H	Dy2	O24	128.35(19)	O4	Dy2	O24	82.0(5)
O1H	Dy2	O25	128.35(19)	O4	Dy2	O31	80.7(9)
O1H	Dy2	O31	71.48(17)	O4	Dy2	O3	81.3(6)
O1H	Dy2	O3	71.48(17)				
O1H2	Dy1	Dy11	42.34(13)				
O1H2	Dy1	Dy2	101.91(14)				

Table S4. Hydrogen bonding interactions (Å. °) of compound 6_{Dy}

D-H···A[a]	***D-H***	***H···A***	***D···A***	***D-H···A***
N1-H1A···O1[1]	0.91	2.10	2.951 (10)	155.8

[1]1-x,1-y,1-z **[a]D: donor. A: acceptor**.

Powder X-ray diffraction analysis

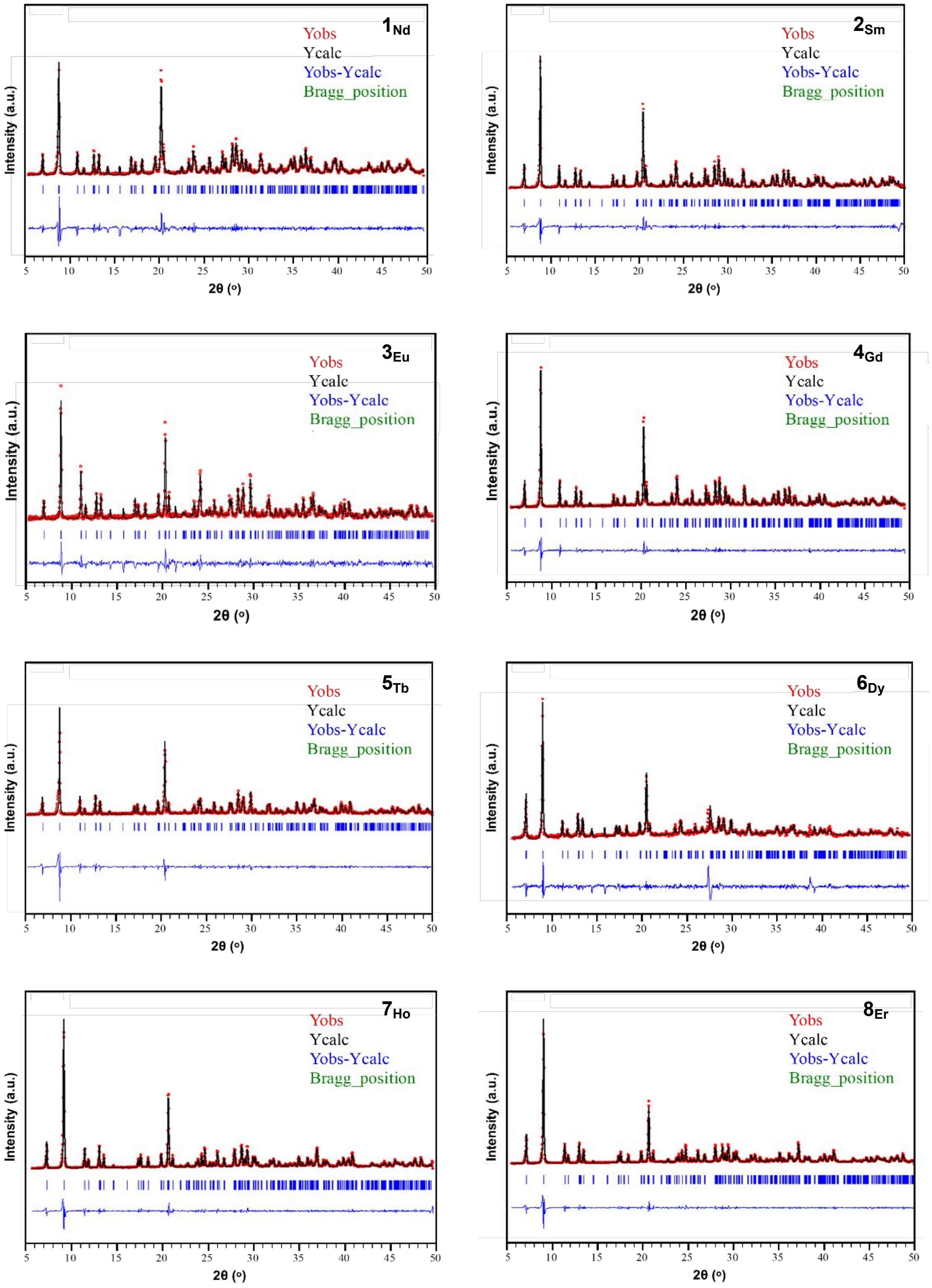

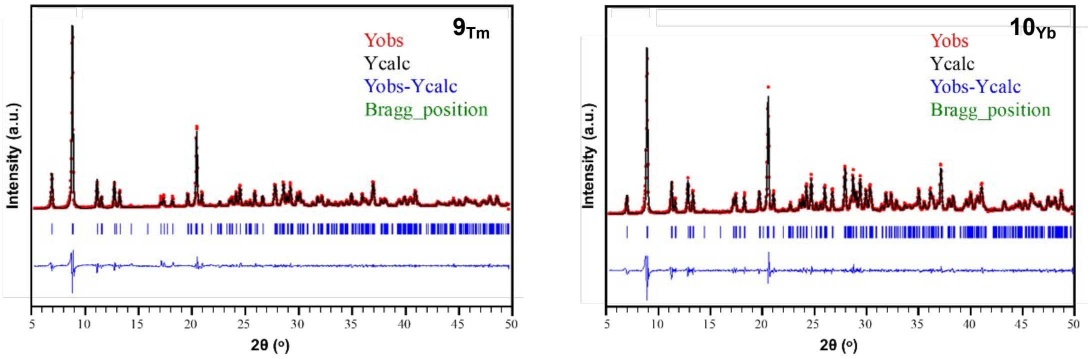

Figure S2. Figure of the pattern matching analysis and experimental PXRD for complexes 1-10.

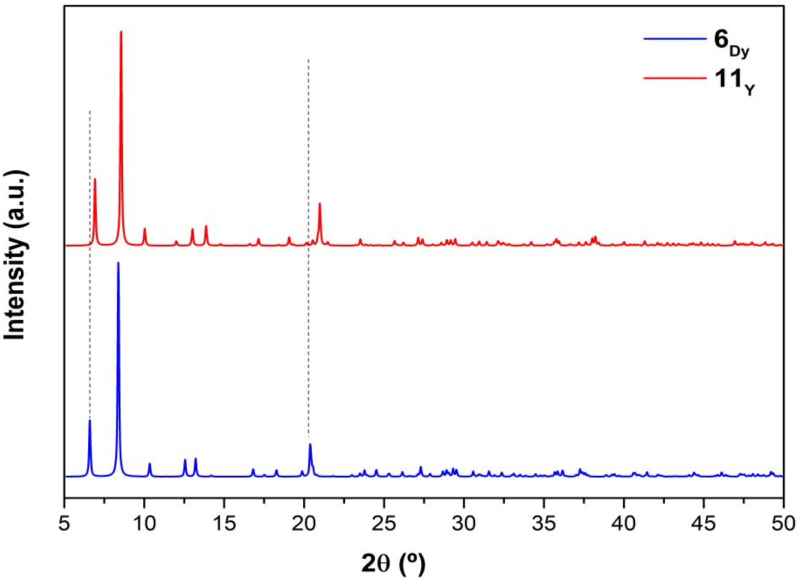

Figure S3. Figure of the theoretical simulated-PXRD for complexes 6_{Dy} and 11_{Y}.

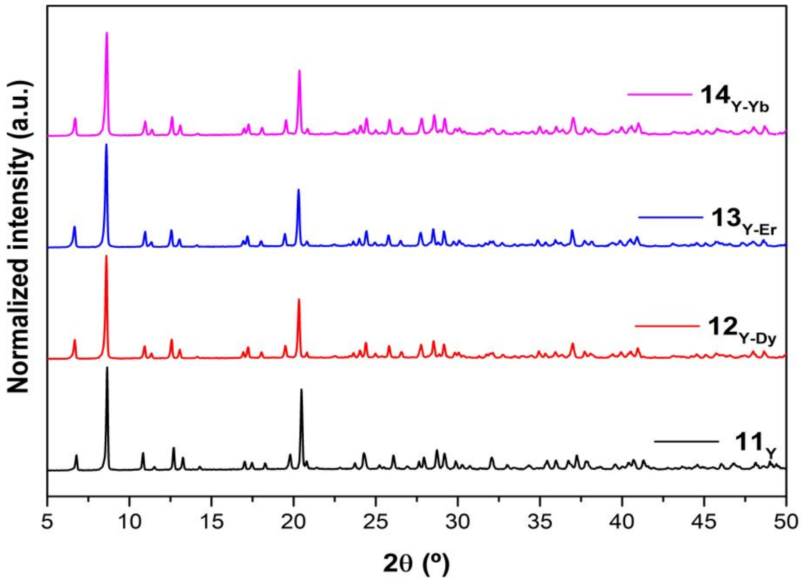

Figure S4.PXRD for complexes 11-14.

When the quaternary compounds (containing yttrium. europium and terbium ions and the ligand) are characterized. it is observed that. depending on the Y^{3+} to Ln^{3+} doping proportion. PXRD patterns present

diffraction maxima corresponding to both pure compounds **11** and **1-10**. This fact. a priori. is indicative of a crystal phase segregation. although it makes no much sense given the isostructural nature of the compounds. Nevertheless. SEM mapping experiments have shown that even if mixture of two type of crystals could happen. the three elements are randomly distributed along a single crystal (for more details see Figure S24).

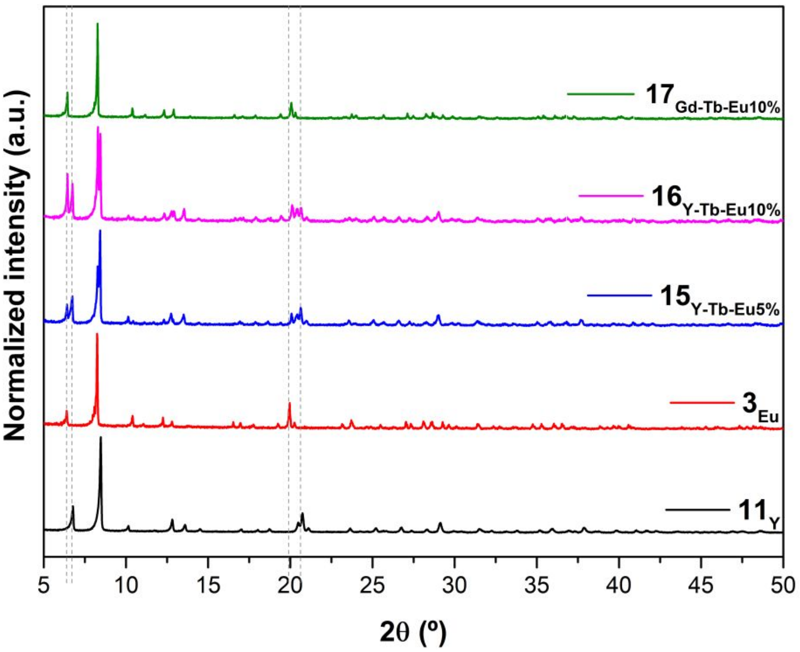

Figure S5. PXRD for complexes 3. 11. 15-17.

Continuous Shape Measurements

CShMs for the coordination environment of compound **6**. The lowest SHAPE values for each ion are shown highlighted in bold. indicating best fits.

Table S5. Table of the continuous Shape Measurements for the LnN_3O_6 coordination environment.

EP-9	1 D9h	Enneagon
OPY-9	2 C8v	Octagonal pyramid
HBPY-9	3 D7h	Heptagonal bipyramid
JTC-9	4 C3v	Johnson triangular cupola J3
JCCU-9	5 C4v	Capped cube J8
CCU-9	6 C4v	Spherical-relaxed capped cube
JCSAPR-9	7 C4v	Capped square antiprism J10
CSAPR-9	8 C4v	Spherical capped square antiprism
JTCTPR-9	9 D3h	Tricapped trigonal prism J51
TCTPR-9	10 D3h	Spherical tricapped trigonal prism
JTDIC-9	11 C3v	Tridiminished icosahedron J63
HH-9	12 C2v	Hula-hoop
MFF-9	13 Cs	Muffin

Complex	JCSAPR-9	CSAPR-9	JTCTPR-9	TCTPR-9	MFF-9
**Dy1 (6)**	2.280	1.243	2.073	**0.890**	1.630

Table S6. Table of the continuous Shape Measurements for the LnO_8 coordination environment.

OP-8	1 D8h	Octagon
HPY-8	2 C7v	Heptagonal pyramid
HBPY-8	3 D6h	Hexagonal bipyramid
CU-8	4 Oh	Cube
SAPR-8	5 D4d	Square antiprism
TDD-8	6 D2d	Triangular dodecahedron
JGBF-8	7 D2d	Johnson - Gyrobifastigium (J26)
JETBPY-8	8 D3h	Johnson - Elongated triangular bipyramid (J14)
JBTP-8	9 C2v	Johnson - Biaugmented trigonal prism (J50)
BTPR-8	10 C2v	Biaugmented trigonal prism
JSD-8	11 D2d	Snub disphenoid (J84)
TT-8	12 Td	Triakis tetrahedron
ETBPY-8	13 D3h	Elongated trigonal bipyramid

Complex	SAPR-8	TDD-8	JBTPR-8	BTPR-8	JSD-8
**Dy2 (6)**	2.973	**2.770**	3.784	3.342	5.612

Thermal analysis

Thermogravimetric analyses have been performed over polycrystalline sample in compound $\mathbf{6_{Dy}}$ in order to check the stability of the product. The TG curves has been collected for compound $\mathbf{6_{Dy}}$ before and after solvent exchange with MeOH. This procedure has been carried out suspending the material in MeOH for 16 h. Solvent exchange procedure has been accomplished as an approach to replace solvent molecules (dimethylformamide and water molecules) to ease material activation to posteriorly analyse its adsorptive-capacity. Powder X-ray diffraction confirmed that $\mathbf{6_{Dy}}$ remains stable after solvent exchange with MeOH as it can be seen in Figure S6. right.

The thermal behaviour of the bulk $[Dy_5L_6(OH)_3(DMF)_3]\cdot 5H_2O$. compound **6**. as synthesised materials were also investigated between ambient temperature and ca. 800 °C in order to study its thermal stability. Due to the isotypical nature of the compounds the following paragraph discussion will be solely focused on compound **6**. Three main regions are appreciable in the TG curve. The first wight loss. between ambient temperature and up to 300 ºC concerns to the progressive loss of solvent molecules. firstly. lattice water molecules and then coordinated DMF molecules. Above this temperature. there is an abrupt descent that corresponds to the collapse of the crystal structure. From the shape of the TG curve. it seems that solvent molecules stabilise the structure and their removal promote crystal structure decomposition. In the final step as a consequence of the decomposition of the organic content metal oxide is obtained

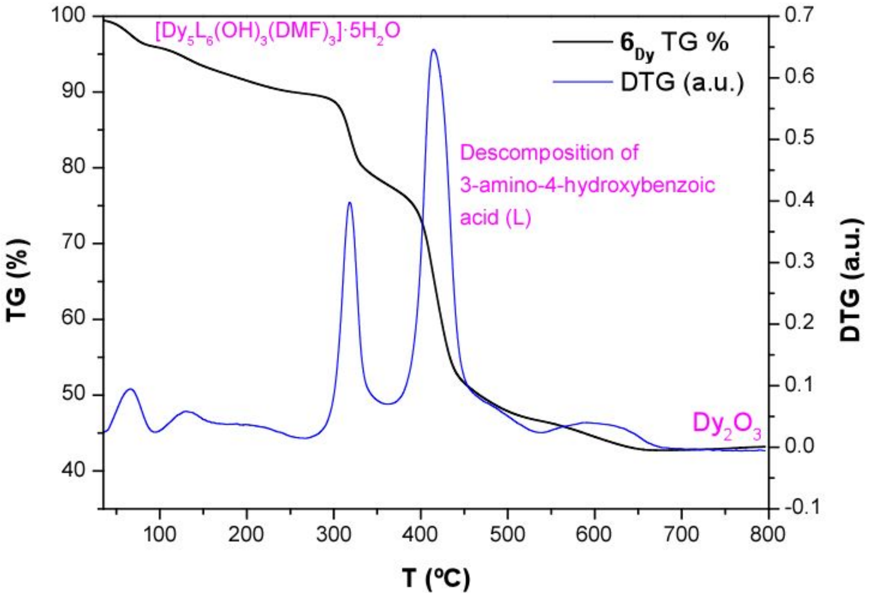

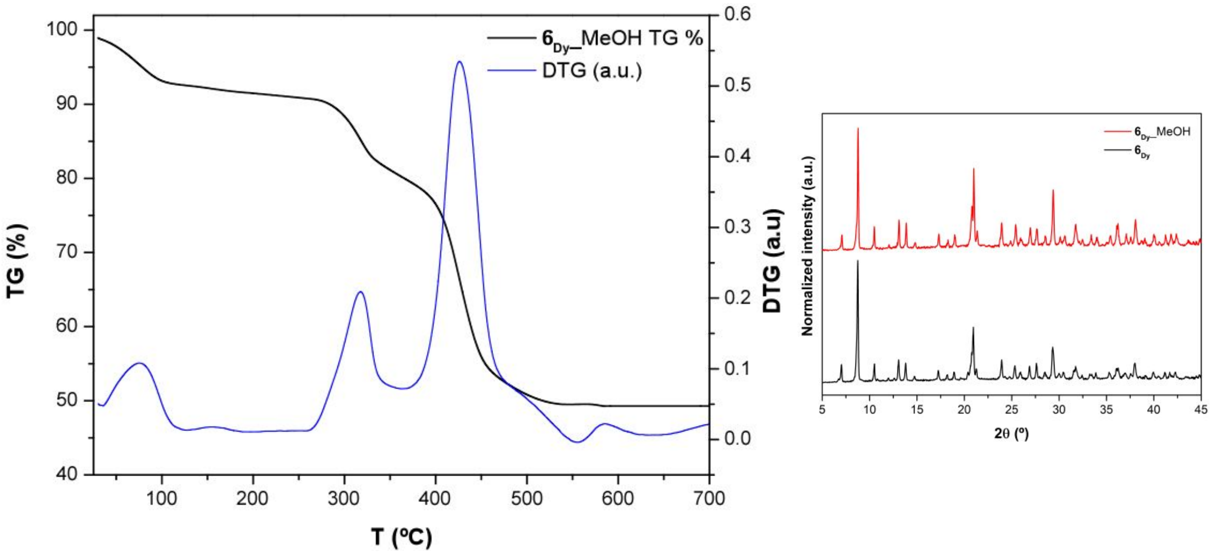

Figure S6. Figure of TG/DTG analysis of compound 6_{Dy} (up. as synthesised). after solvent exchange with MeOH during 16 h (down-left) and figure of the experimental PXRD for complex 6_{Dy} before and after solvent exchange with MeOH (down-right).

After solvent exchange with MeOH. TG curve performed in **6_{Dy}_**MeOH shows a plateau at around 100 ºC suggesting that solvent molecules (DMF and water) have been properly replaced with MeOH which would evaporate up to the indicated temperature. Around 100-200 ºC it seems that the MOF desolvatated skeleton is obtained. Finally. as another evidence for the proper solvent exchange. final-residue Dy_2O_3 percentage has increased by 6 %. It is expected since DMF and water molecules are heavier and have higher boiling points than MeOH.

Thermal evolution

Thermal evolution of compound $\mathbf{6_{Dy}}$ shows that the compound maintains its crystallinity up to 230 ^{o}C. These results come in line with thermogravimetric analysis. where it could be seen that above this temperature structure collapses and evolves into the metallic residue Dy_2O_3 at around 800 ^{o}C

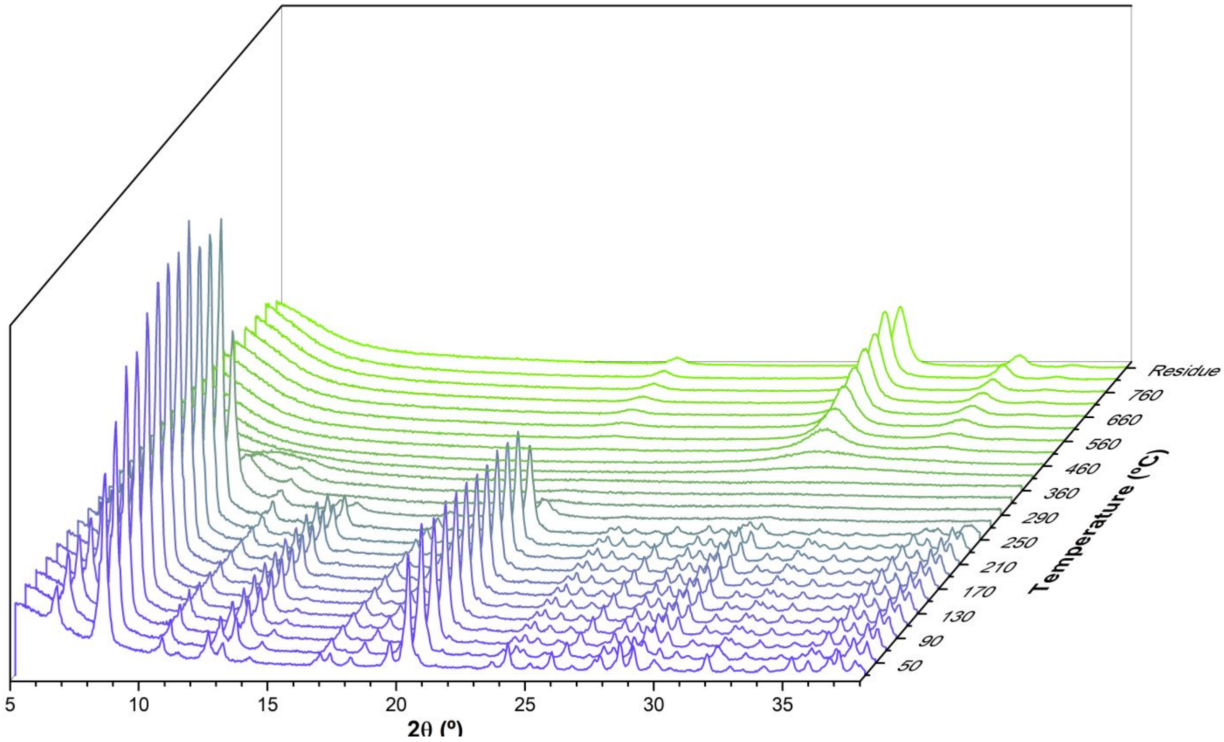

Figure S7. Thermal evolution of compound $\mathbf{6_{Dy}}$.

Additional views of the structure

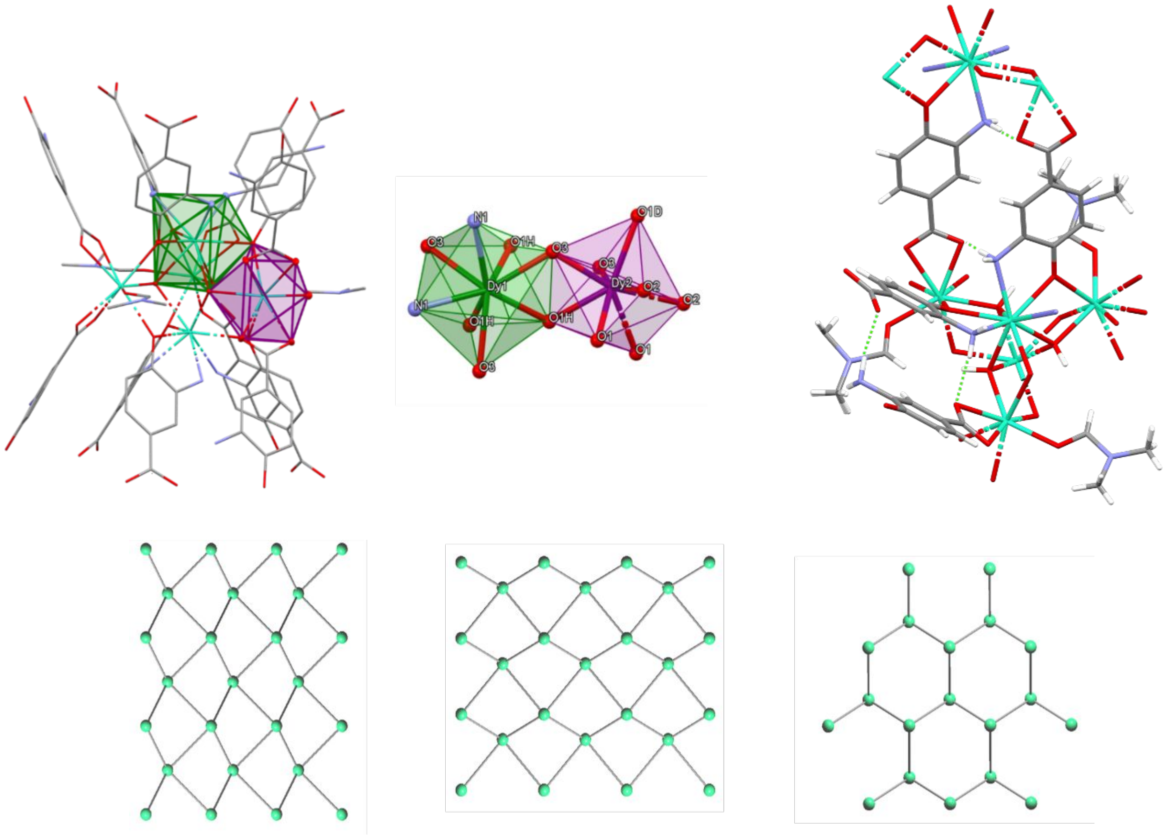

Compound 6

Figure S8. View of pentametallic nodous showing Dy1 and Dy2 coordination polyhedra (left and middle. up). the most representative intermolecular interactions exhibiting hydrogen bonds highlighted in light green (right. up) and the topological representation along *a*. *b* and *c* axis (down)

Ac magnetic susceptibility measurements

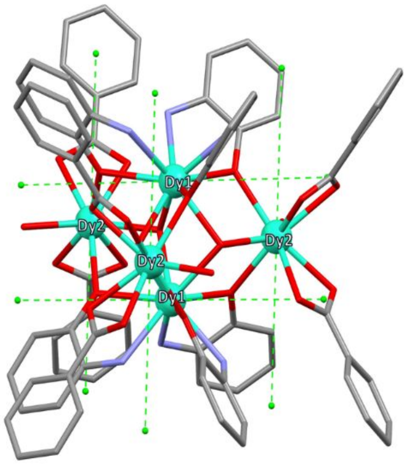

Figure S9. Magnetic axes of the Dy^{III} ions calculated with the Magellan software[7]

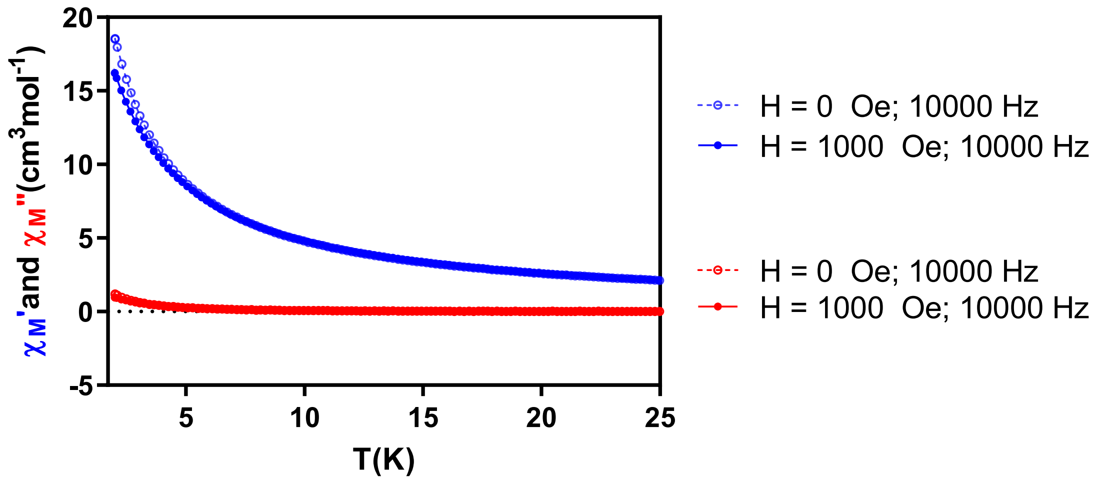

Figure S10. Temperature dependence of in-phase (red) and out-of-phase (blue) components of the ac susceptibility in a zero applied dc field for 6_{Dy}

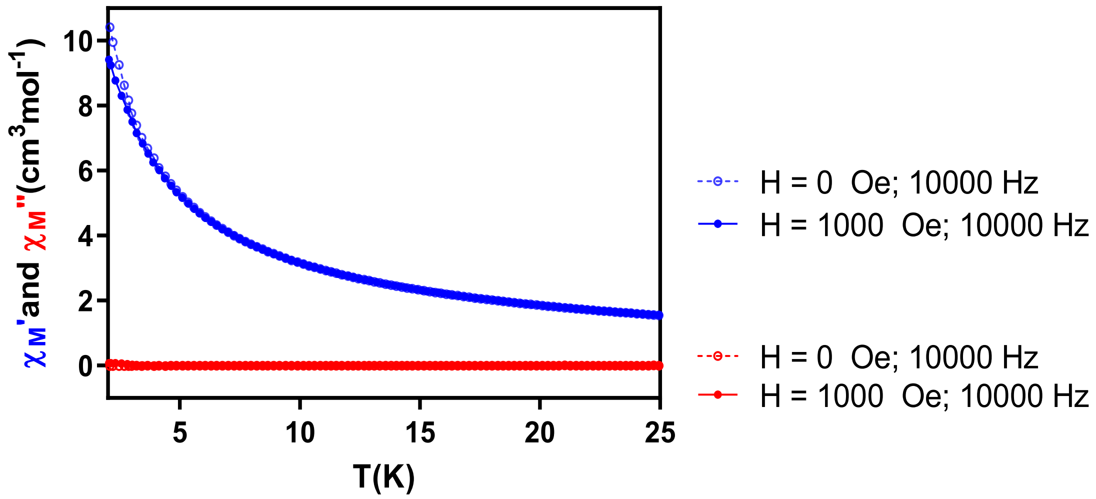

Figure S11. Temperature dependence of in-phase (red) and out-of-phase (blue) components of the ac susceptibility in a zero applied dc field for 8_{Er}

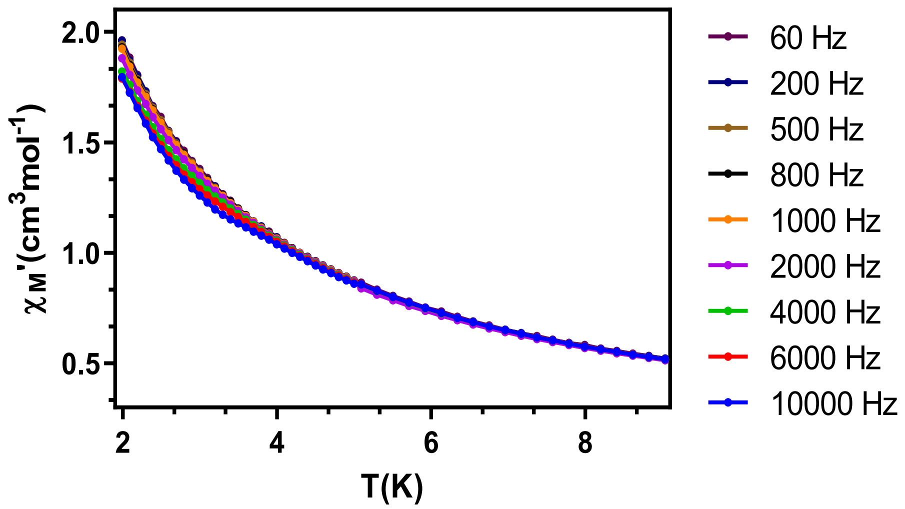

Figure S12. Temperature dependence of in-phase components of the *ac* susceptibility in a *dc* applied field of 1000 Oe for 10_{Yb}

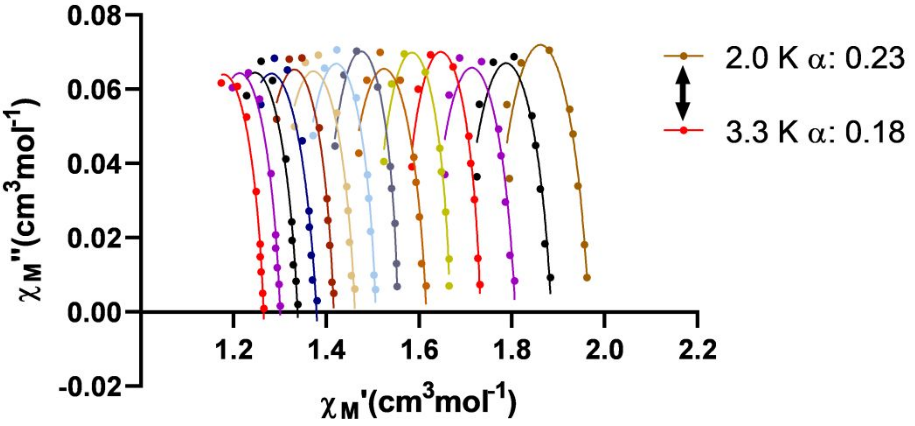

Figure S13. Cole-Cole plots in a *dc* applied field of 1000 Oe for 10_{Yb}

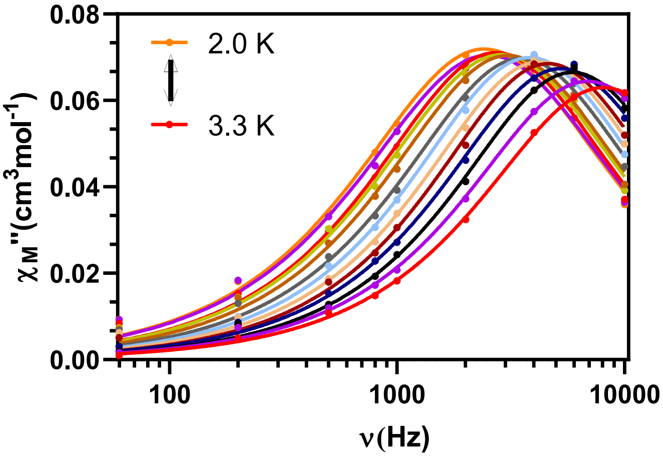

Figure S14. Variable-temperature frequency dependence of the χM'' signal under 1000 Oe applied field for 10_{Yb}. Solid lines represent the best fitting of the experimental data to the Debye model.

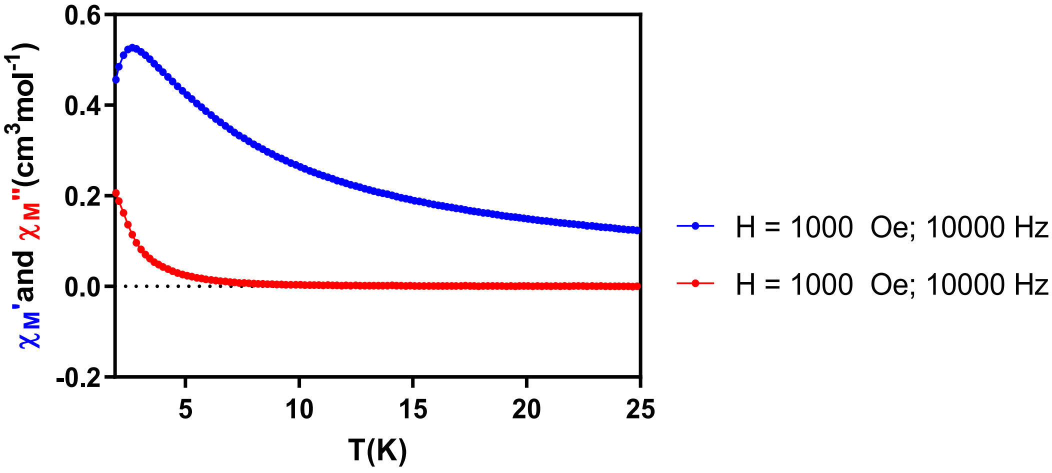

Figure S15. Temperature dependence of in-phase (blue) and out of phase (red) components of the *ac* susceptibility in a *dc* applied field of 1000 Oe for $12_{Y\text{-}Dy}$

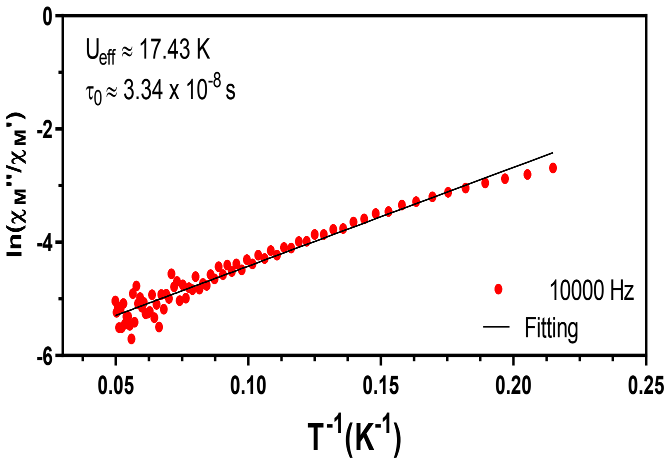

Figure S16. Plot of ln(χ_M''/χ_M') versus 1/T at 10000 Hz for compound 12$_{Y\text{-}Dy}$ under an applied field of 1000 Oe. The solid lines represent the linear fit with ln(χ_M''/χ_M') = ln($2\pi\nu\tau_0$)+ E_a/k_BT.

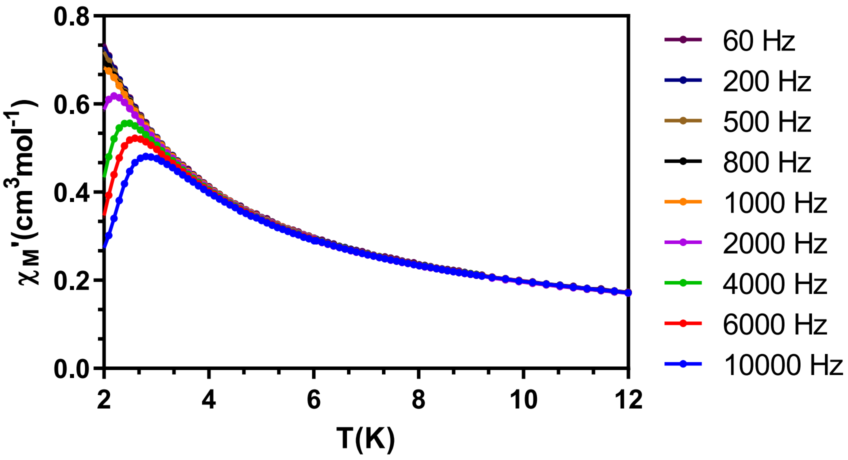

Figure S17. Temperature dependence of in-phase components of the *ac* susceptibility in a *dc* applied field of 1000 Oe for 13$_{Y\text{-}Er}$

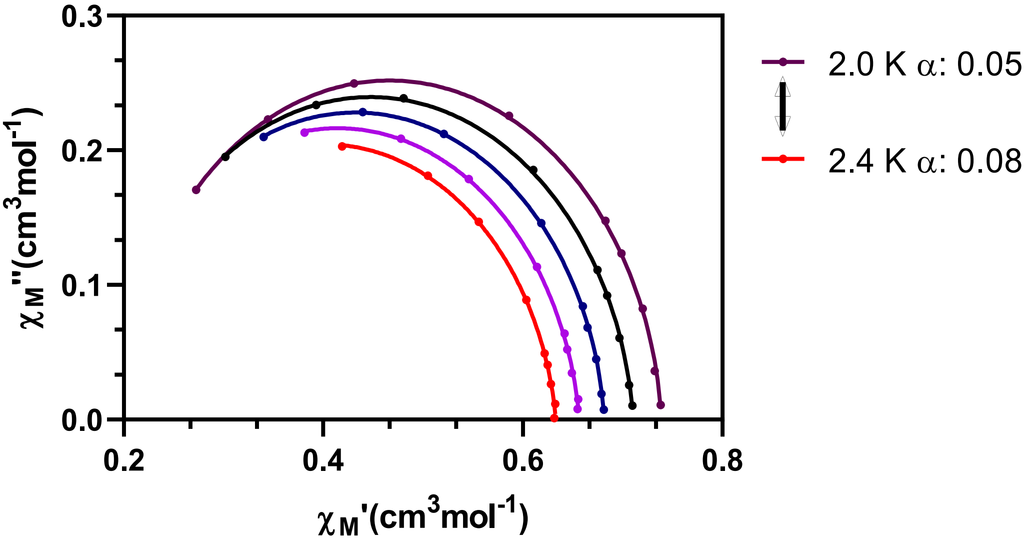

Figure S18. Cole-Cole plots in a *dc* applied field of 1000 Oe for $13_{Y\text{-}Er}$

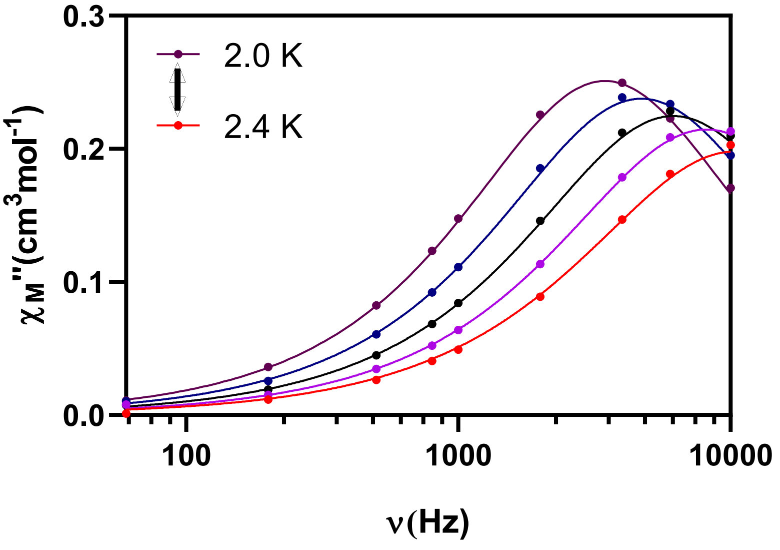

Figure S19. Variable-temperature frequency dependence of the χM" signal under 1000 Oe applied field for $13_{Y\text{-}Er}$. Solid lines represent the best fitting of the experimental data to the Debye model.

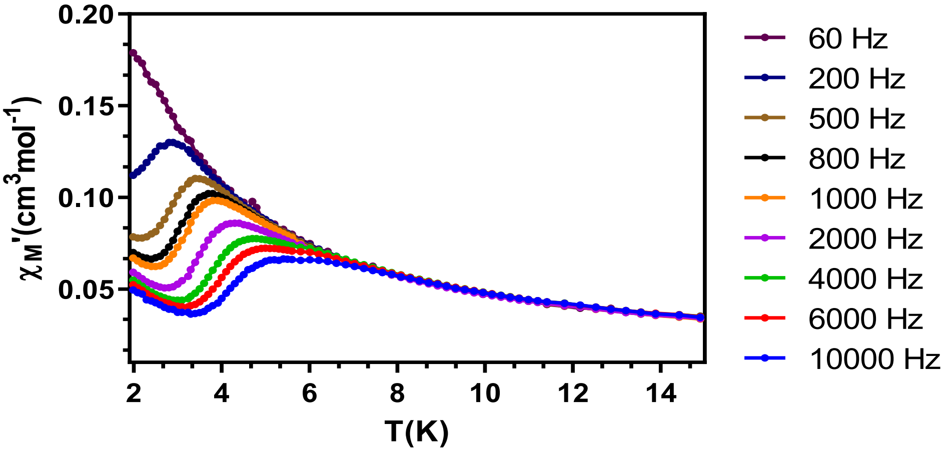

Figure S20. Temperature dependence of in-phase components of the *ac* susceptibility in a *dc* applied field of 1000 Oe for $\mathbf{14_{Y\text{-}Yb}}$

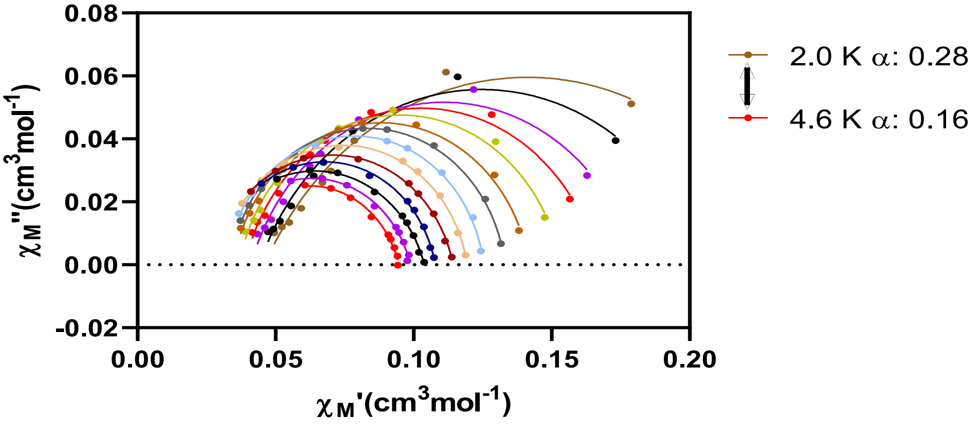

Figure S21. Cole-Cole plots in a *dc* applied field of 1000 Oe for $\mathbf{14_{Y\text{-}Yb}}$

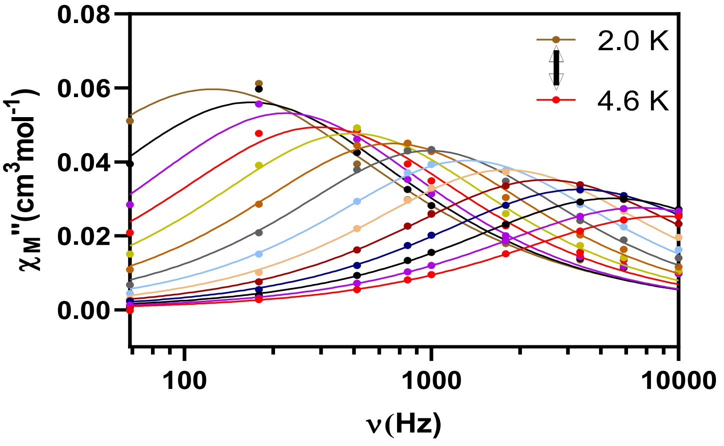

Figure S22. Variable-temperature frequency dependence of the χ_M'' signal under 1000 Oe applied field for $\mathbf{14_{Y\text{-}Yb}}$. Solid lines represent the best fitting of the experimental data to the Debye model.

Scanning Electron Microscopy

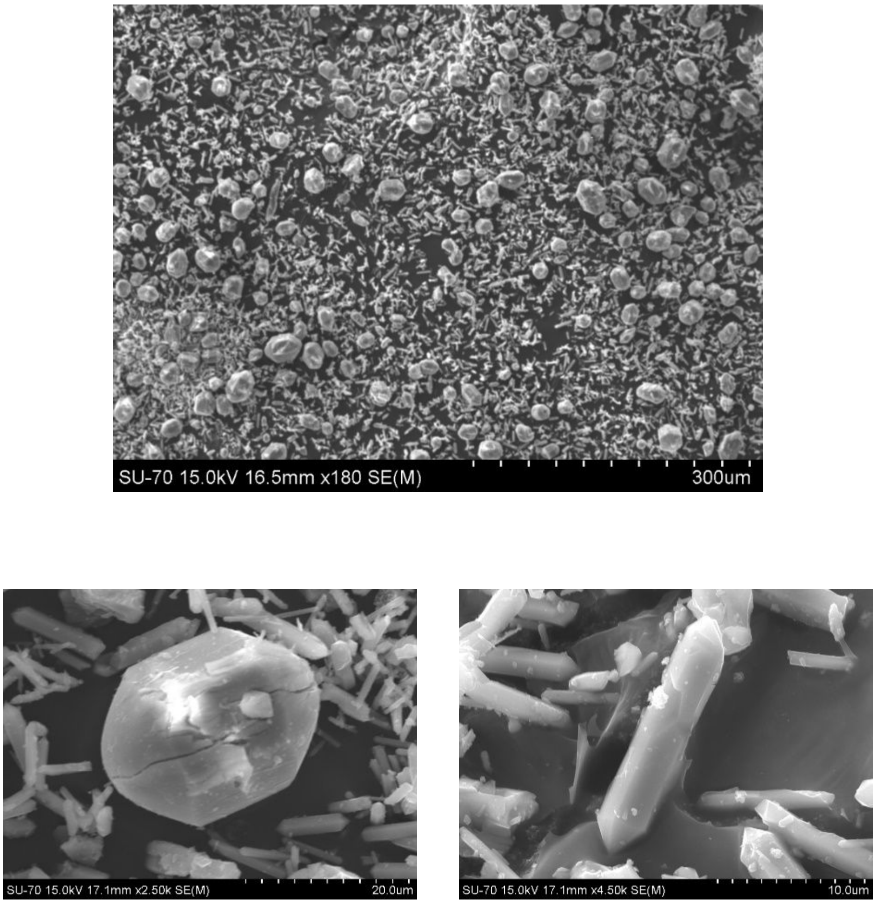

Figure S23. SEM images of compound $16_{Y\text{-}Tb\text{-}Eu10\%}$ where two types of crystal are distinguished.

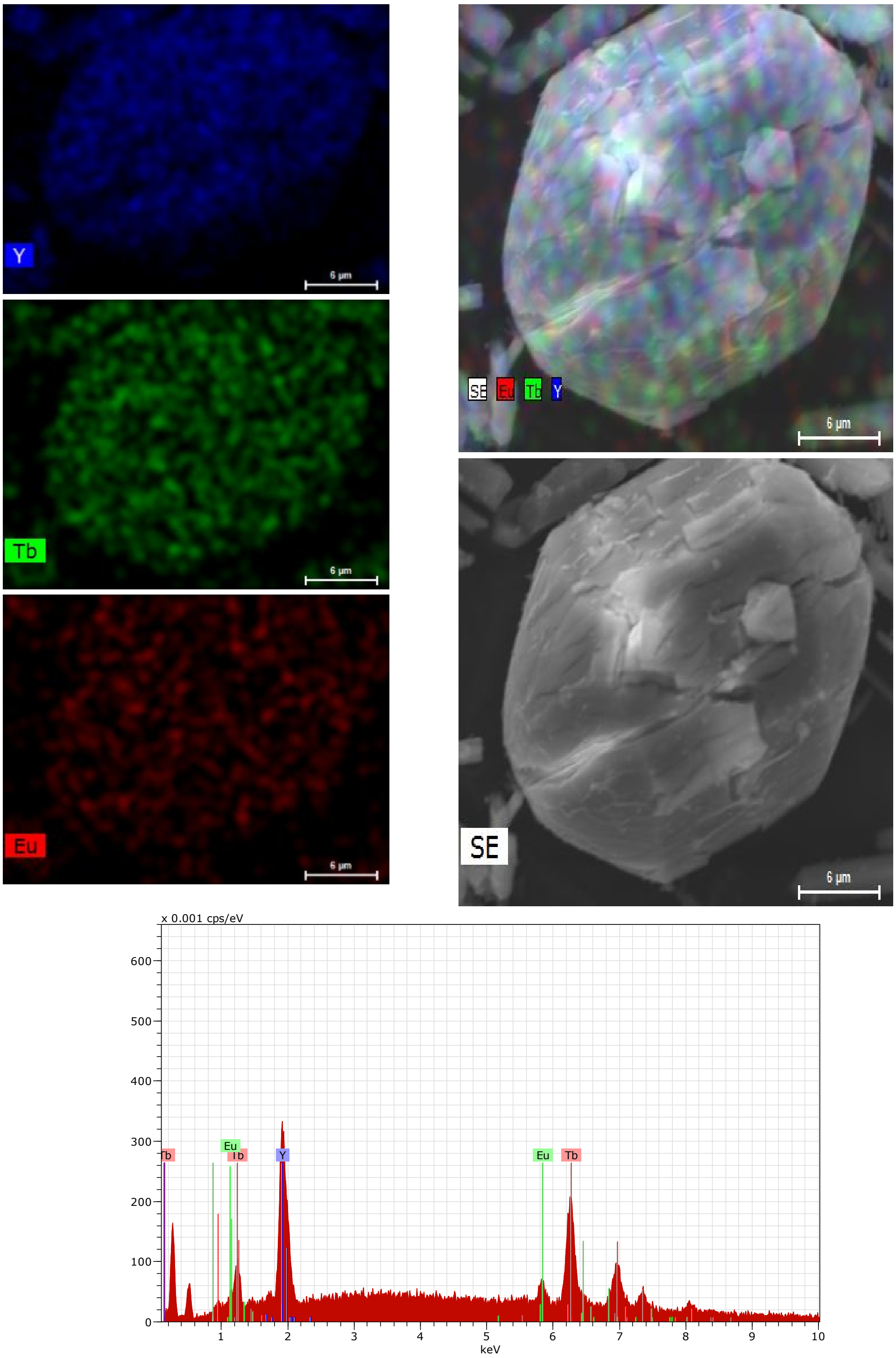

Spectrum: crystal type 1

Element	Series	unn. C [wt.%]	norm. C [wt.%]	Atom. C [at.%]	Error (3 Sigma) [wt.%]
Terbium	L-series	37.80	56.13	**43.91**	3.78
Europium	L-series	6.10	9.05	**7.41**	0.84
Yttrium	L-series	23.44	34.81	**48.68**	3.04
	Total:	67.34	100.00	100.00	

Figure S24. SEM image. EDS spectrum and elemental quantitative data representative of the crystal type 1 of compound $16_{Y\text{-}Tb\text{-}Eu10\%}$.

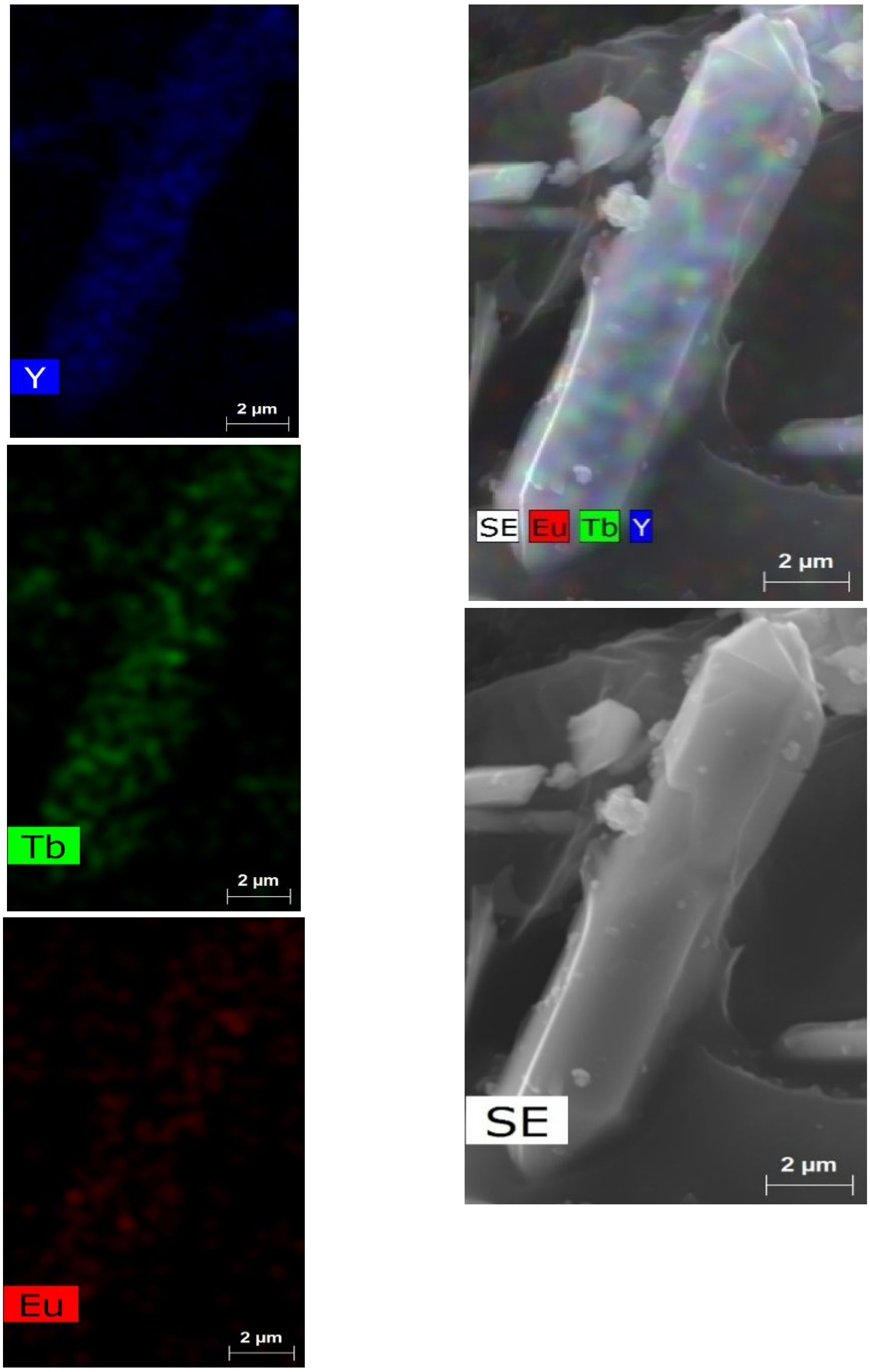
Y
2 µm
Tb
2 µm
Eu
2 µm
SE Eu Tb Y
2 µm
SE
2 µm

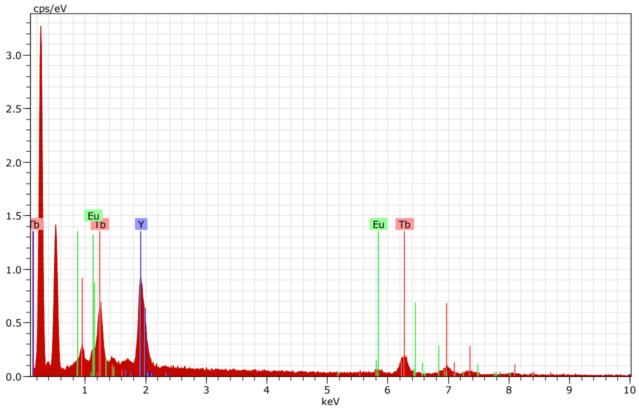

Spectrum: crystal type 2

Element	Series	unn. C [wt.%]	norm. C [wt.%]	Atom. C [at.%]	Error (3 Sigma) [wt.%]
Terbium	L-series	39.37	55.08	**42.75**	4.18
Europium	L-series	6.29	8.81	**7.15**	1.01
Yttrium	L-series	25.81	36.11	**50.10**	3.23
	Total:	71.47	100.00	100.00	

Figure S25. SEM image. EDS spectrum and elemental quantitative data representative of the crystal type 2 of compound $16_{Y\text{-}Tb\text{-}Eu10\%}$.

Determination of the metal content by ICP-AES

Table S7. ICP-AES results of compounds 15-17

	Content			Percentage		
**Compounds**	Yor Gd mg L^{-1} (mM)	Tb mg L^{-1} (mM)	Eu mg L^{-1} (mM)	Y or Gd %	Tb %	Eu%
**$15_{Y-Tb-Eu5\%}$**	491 mg L^{-1} (5.52 mM)	869 mg L^{-1} (5.47 mM)	73.8 mg L^{-1} (0.49 mM)	48.1	47.7	4.2
**$16_{Y-Tb-Eu10\%}$**	463 mg L^{-1} (5.20 mM)	756 mg L^{-1} (4.75 mM)	132 mg L^{-1} (0.87 mM)	48.1	43.9	8.0
**$17_{Gd-Tb-Eu10\%}$**	838 mg L^{-1} (5.33 mM)	746 mg L^{-1} (4.69 mM)	142 mg L^{-1} (0.93 mM)	48.6	42.9	8.5

Photoluminescence measurements

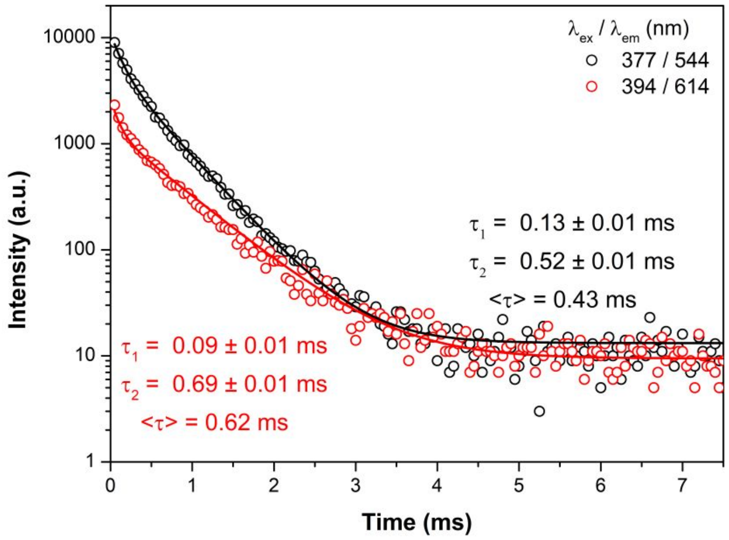

Figure S26. 12 K Decay curves monitoring the emission at 544 nm and 614 nm with the excitation selected at 377 nm (black), and 394 nm (red); the solid lines are the best fits using second-order decay functions, $y = y_0 + A_1 \cdot exp(-x/\tau_1) + A_2 \cdot exp(-x/\tau_2)$ ($r^2 > 0.999$). The average lifetime was defined as $<\tau> = (A_1\tau_1^2 + A_2\tau_2^2)/(A_1\tau_1 + A_2\tau_2)$.

Heterometallic compound **$15_{Y-Tb-Eu5\%}$** decay curves collected at 12 K have been acquired by monitoring the strongest emission lines for both Tb^{3+} ($^5D_4 \rightarrow {}^7F_5$ transition, 544 nm) and Eu^{3+} ($^5D_0 \rightarrow {}^7F_2$ transition, 614 nm), using the excitations at 377 nm and 394 nm, respectively (Figure S26). All curves are well fitted by a second order exponential decay functions yielding average lifetimes: of 0.43 ms and 0.62 ms, respectively for Tb^{3+} and Eu^{3+}, in one hand, both lifetimes are larger than the ones obtained for the pure Tb^{3+} (**5_{Tb}**) and Eu^{3+} (**3_{Eu}**) samples, resulting from the suppression of the self-quenching due to the dilution of the optically active elements. On the other hand, contrary to the pure Tb^{3+} and Eu^{3+} samples, the Eu^{3+} lifetimes are larger than the ones of Tb^{3+}, due to the aforementioned Tb^{3+}-to-Eu^{3+} energy transfer process.

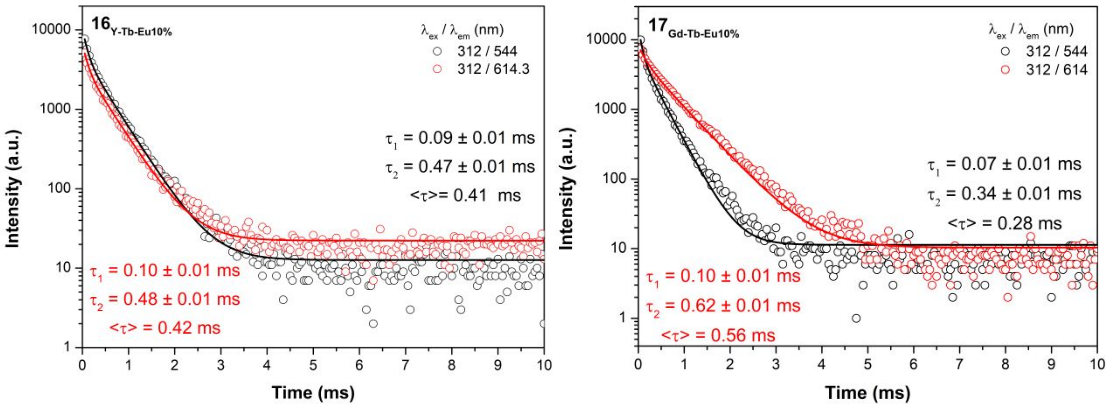

Figure S27. Decay curves of $\mathbf{16_{Y\text{-}Tb\text{-}Eu10\%}}$ (left) and $\mathbf{17_{Gd\text{-}Tb\text{-}Eu10\%}}$ (right) acquired at 12 K monitoring the Tb^{3+}emission at 544 nm (black) and the Eu^{3+}emission at 614 nm (red) with the excitation fixed at 312 nm. The solid lines are the best fits using second order exponential decay function $y = y_0 + A_1 \cdot \exp(-x/\tau_1) + A_2 \cdot \exp(-x/\tau_2)$ ($r^2 > 0.999$). Average lifetime was calculated according to the formula $<\tau> = (A_1\tau_1^2 + A_2\tau_2^2)/(A_1\tau_1 + A_2\tau_2)$.

Finally, $^5D_4$ Tb^{3+} and $^5D_0$ Eu^{3+} decay curves, monitoring the emissions at 544 and 614 nm, respectively, were collected for compounds $\mathbf{16_{Y\text{-}Tb\text{-}Eu10\%}}$ and $\mathbf{17_{Gd\text{-}Tb\text{-}Eu10\%}}$ at 12 K with the excitation fixed at 312 nm (**Error! Reference source not found.**). The average lifetimes shows that compound $\mathbf{16_{Y\text{-}Tb\text{-}Eu10\%}}$ ($<\tau_{544\,(\mathbf{16})}> = 0.41$ ms, $<\tau_{614(\mathbf{16})}> = 0.42$ ms) presents the larger Tb^{3+} lifetimes and the shorter Eu^{3+} lifetimes relatively to compound $\mathbf{17_{Gd\text{-}Tb\text{-}Eu10\%}}$ ($<\tau_{544\,(\mathbf{17})}> = 0.28$ ms, $<\tau_{614\,(\mathbf{17})}> = 0.56$ ms). In both cases, lifetimes increase relatively to the values obtained at 12 K for the pure $\mathbf{3_{Eu}}$ ($<\tau> = 0.27$ ms) and $\mathbf{5_{Tb}}$ ($<\tau> = 0.29$ ms) compounds. This results from the Tb^{3+}-to-Eu^{3+} energy transfer, that shortens the Tb^{3+} lifetimes and increases the Eu^{3+} lifetimes, and from the dilution of the optically active ions, that eliminates the self-quenching effect.

Table S8. Representative cryogenic lanthanide-bearing luminescent ratiometric thermometers (Sm is the maximum relative sensitivity at temperature Tm. ΔT is the temperature uncertainty with a relative sensitivity Sr > 1.0 %K^{-1} in a given temperature range)

Materials	S_m (% K^{-1})	T_m [K]	ΔT [K] (S_r>1.0 %K^{-1})	Ref.
$Sr_2GeO_4:Pr^{3+}$	9.0	22	17–92	8
$Sr_2(Ge.Si)O_4:Pr^{3+}$	9.2	65	50–95	9
LaF_3:2.96 % Er^{3+}.1.57 % Yb^{3+}	6.6	15	15–45	10
MoO_3:Tb.Eu	9.2	15	15–67	11
$Na_2K[(Lu_{0.75}Yb_{0.20}Er_{0.05})_3Si_6O_{18}]$	2.60	27	12–86	12,13
$[Tb_{3.94}Eu_{0.06}(MoO_4)(Mo_7O_{24})_4]^{14-}$	4.76	50	18–100	14
$Tb_{0.95}Eu_{0.05}HY^a$	31.0	4	4–48	15
$Eu_{0.02}Gd_{0.98}(dsb)^b$	7.14	65	20–35; 65–145	16
$[(Tb_{0.914}Eu_{0.086})(pda)_3(H_2O)]\cdot 2H_2O^c$	5.96	25	12–80	17
$[Eu_{0.102}Tb_{0.898}(notpH_4)(NO_3)(H_2O)]\cdot 8H_2O^d$	3.90	38	18–245	18
$Tb_{0.95}Eu_{0.05}(btb)^e$	2.85	14	14–177	19
$[Tb_{0.9}Eu_{0.1}(1.3\text{-}bdc)_3(H_2O)_2]\cdot H_2O^f$	3.30	36	12–101	20
$Na[(Gd_{0.82}Tb_{0.14}Eu_{0.04})SiO_4]\cdot xNaOH$	1.9	14	12–30	21
$Na[(Gd_{0.66}Tb_{0.30}Eu_{0.04})SiO_4]\cdot xNaOH$	6.2	12	12–47	21
$[(Y_{0.50}Tb_{0.40}Eu_{0.10})_5L_6(OH)_3(DMF)_3]\cdot 5H_2O$	1.20	12	12–15	This work
$[(Gd_{0.50}Tb_{0.40}Eu_{0.10})_5L_6(OH)_3(DMF)_3]\cdot 5H_2O$	2.43	320	243–300	This work

^{a} H_4Y: 5-hydroxy-1.2.4-benzenetricarboxylic acid. ^{b} H_3dsb: 3.5-disulfobenzoate. ^{c} H_2pda: 1.4-phenylenediacetic acid; H_6notp: 1.4.7-triazacyclononane-1.4.7-triyl-tris(methylenephosphonic acid). ^{d} H_3btb: 1.3.5-tris-(4-carboxyphenyl)benzene. ^{e} H_3btb: 1.3.5-tris-(4-carboxyphenyl)benzene. ^{f} H_2bdc: 1.3-benzene-dicarboxylic acid.

Adsorption properties

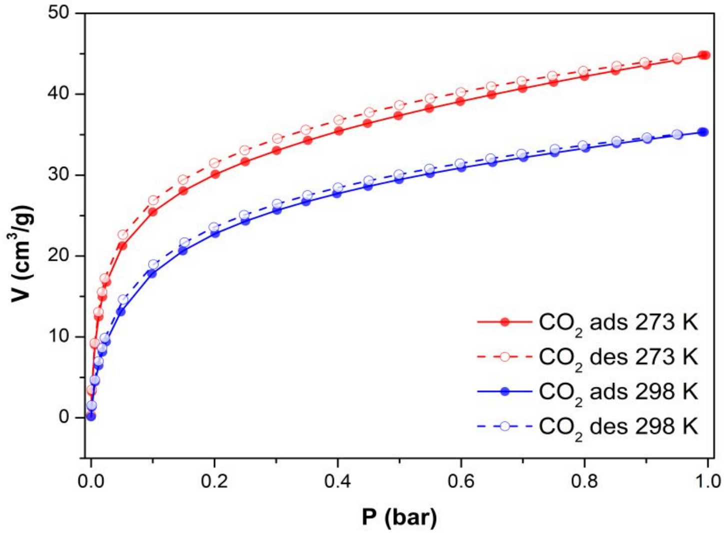

Figure S28 isotherm of compound 6_{Dy} in cm^3/g. The conversion to mmol/g has done taking into account that 1 mmol of any gas at stp (standard temperature and pressure conditions. according to the IUPAC. at 273 K and 1 bar pression conditions) occupies 22.414 cm^3 volume.

The isosteric heat of adsorption (Q_{st}) was calculated from the adsorption isotherms at 273 and 298 K to evaluate the strength of the interaction between the adsorbent and adsorbate according to Clausius-Clapeyron equation, Equation **1**.[22]

$$Q_{st} = -R\,[\Delta(lnP)/\Delta(T^{-1})]_N \qquad \text{Equation } \mathbf{1}$$

where Q_{st}, R, P, T and N correspond to isosteric heat of adsorption, constant for ideal gases, pressure, temperature and the amount of adsorbed CO_2, respectively. Enthalpy of adsorption (Q_{st}) value demonstrates the strength of the interaction between the host and guest molecules. Indeed, the magnitude of the Q_{st} is a function of the binding strength indicating the amount of the required energy for the regeneration process. The higher Q_{st} of adsorption, the higher will be the affinity that the adsorbent has to adsorb a gas, CO_2 in this case. Nevertheless, its regeneration process would require high energy to regenerate the adsorbent. Thus, an equilibrium or balance is needed between the affinity that MOFs has to uptake a gas a specific gas, the enthalpy of adsorption, and the energy required to regenerate the material.[23]

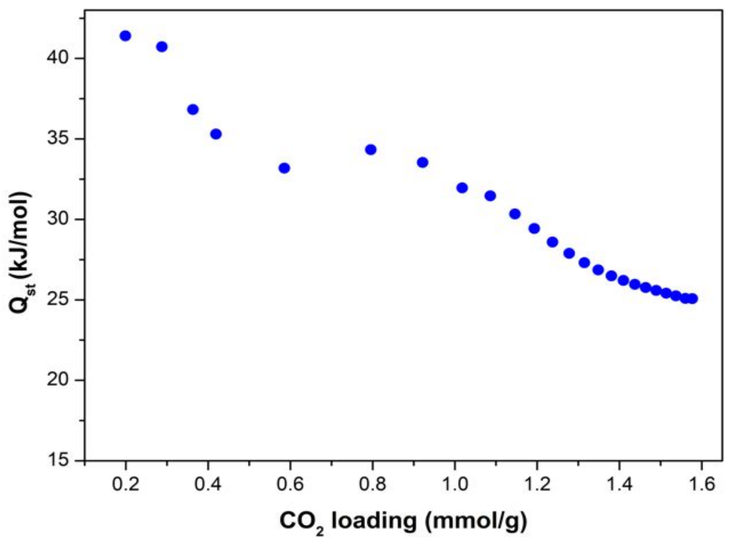

Figure S29. Isosteric heats of adsorption (Q_{st}) of CO_2 per Dy_5 cluster for compound 6_{Dy}.

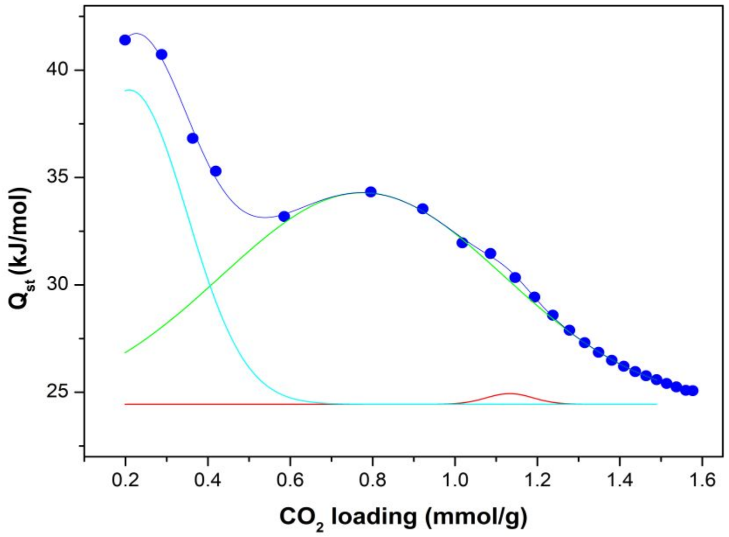

Figure S30. Deconvolution of isosteric heat of adsorption (Q_{st}) of CO_2 for compound 6_{Dy}

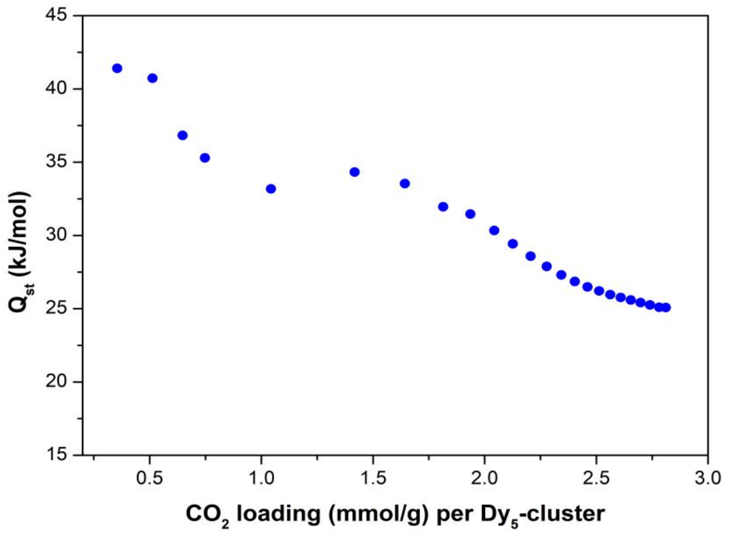

Figure S31. Isosteric heat of adsorption of CO_2 loading (mmol/g) per Dy_5 cluster.

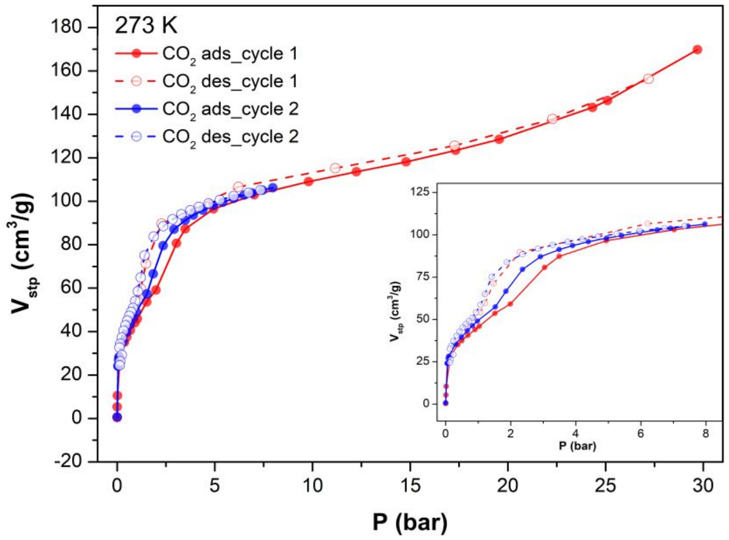

Figure S32. High pressure CO_2 adsorption-desorption regeneration cycles for compound 6 at 273 K.

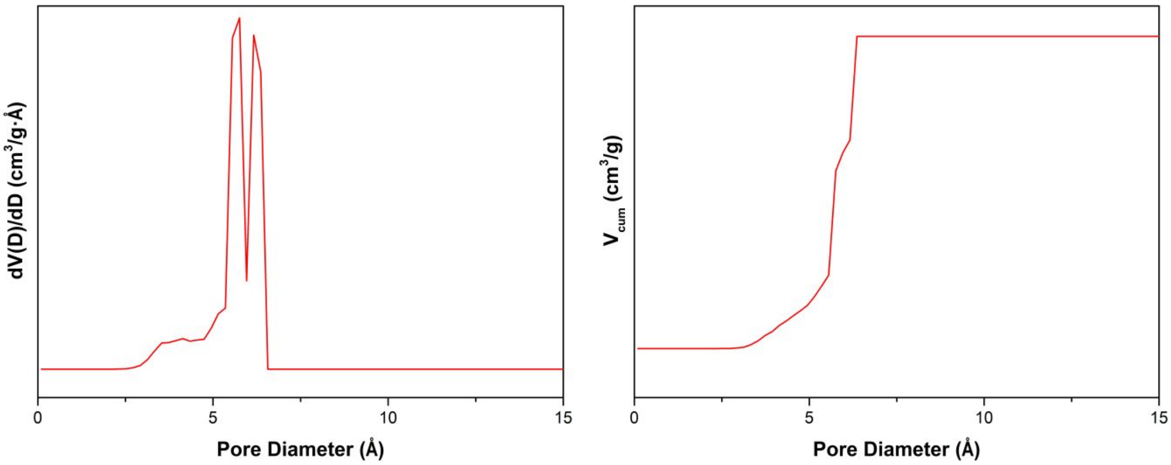

Figure S33. Derivative Geometric Pore Volume (pore size distribution) and cumulative volume in anhydrous compound 6

Table S9. Calculations performed by Monte Carlo procedure.[24,25]

Compound	Area & Vol			Pore diameter		
	S_{area} ($m^2 \cdot g^{-1}$)	V_{pore} ($cm^3 \cdot g^{-1}$)	Porosity (%)	Limiting (Å)	Maximun (Å)	Dimensionality
6_{Dy} anhydrous	713.2	0.319	50.9	4.89	6.35	3 D

References

(1) A. Earnshaw. *Introduction to Magnetochemistry*, Academic P.; London, 1968.

(2) Sheldrick, G. M. SADABS. University of Gottingen: Gottingen, Germany 1996, p Program for Empirical Adsorption Correction.

(3) Altomare, A.; Burla, M. C. M. C.; Camalli, M.; Cascarano, G. L. G. L.; Giacovazzo, C.; Guagliardi, A.; Moliterni, A. G. G. A. G. G.; Polidori, G.; Spagna, R.; IUCr. SIR97: A New Tool for Crystal Structure Determination and Refinement. *urn:issn:0021-8898* **1999**, *32* (1), 115–119. https://doi.org/10.1107/S0021889898007717.

(4) Farrugia, L. J. WinGX and ORTEP for Windows: An Update. *J. Appl. Crystallogr.* **2012**, *45* (4), 849–854. https://doi.org/10.1107/S0021889812029111.

(5) Farrugia, L. J.; IUCr. WinGX Suite for Small-Molecule Single-Crystal Crystallography. *urn:issn:0021-8898* **1999**, *32* (4), 837–838. https://doi.org/10.1107/S0021889899006020.

(6) J. Rodríguez-Carvajal. FULLPROF 2000,Version 2.5d,. *FULLPROF 2000,version 2.5d, Laboratoire Léon Brillouin (CEA-CNRS), Centre d'Études de Saclay, Gif sur Yvette Cedex, France,*. 2000.

(7) Chilton, N. F.; Collison, D.; McInnes, E. J. L. L.; Winpenny, R. E. P. P.; Soncini, A. An Electrostatic Model for the Determination of Magnetic Anisotropy in Dysprosium Complexes. *Nat. Commun.* **2013**, *4* (1), 1–7. https://doi.org/10.1038/ncomms3551.

(8) Brites, C. D. S.; Fiaczyk, K.; Ramalho, J. F. C. B.; Sójka, M.; Carlos, L. D.; Zych, E. Widening the Temperature Range of Luminescent Thermometers through the Intra- and Interconfigurational Transitions of Pr3+. *Adv. Opt. Mater.* **2018**, *6* (10), 1701318. https://doi.org/10.1002/adom.201701318.

(9) Sójka, M.; Ramalho, J. F. C. B.; Brites, C. D. S.; Fiaczyk, K.; Carlos, L. D.; Zych, E. Bandgap Engineering and Excitation Energy Alteration to Manage Luminescence Thermometer Performance. The Case of $Sr_2(Ge,Si)O_4:Pr^{3+}$. *Adv. Opt. Mater.* **2019**, *7* (23), 1901102. https://doi.org/10.1002/ADOM.201901102.

(10) Kaczmarek, A. M.; Kaczmarek, M. K.; Deun, R. Van. Er^{3+}-to-Yb^{3+} and Pr^{3+}-to-Yb^{3+} Energy Transfer for Highly Efficient near-Infrared Cryogenic Optical Temperature Sensing. *Nanoscale* **2019**, *11* (3), 833–837. https://doi.org/10.1039/C8NR08348G.

(11) Liu, J.; Deun, R. Van; Kaczmarek, A. M. Eu^{3+}, Tb^{3+}- and Er^{3+}, Yb^{3+}-Doped α-MoO_3 Nanosheets for Optical Luminescent Thermometry. *Nanomater. 2019, Vol. 9, Page 646* **2019**, *9* (4), 646. https://doi.org/10.3390/NANO9040646.

(12) Ananias, D.; Almeida Paz, F. A.; Carlos, L. D.; Rocha, J. Near-Infrared Ratiometric Luminescent Thermometer Based on a New Lanthanide Silicate. *Chem. - A Eur. J.* **2018**, *24* (46), 11926–11935. https://doi.org/10.1002/chem.201802219.

(13) Ananias, D.; Paz, F. A. A.; Yufit, D. S.; Carlos, L. D.; Rocha, J. Photoluminescent Thermometer Based on a Phase-Transition Lanthanide Silicate with Unusual Structural Disorder. *J. Am. Chem. Soc.* **2015**, *137* (8), 3051–3058. https://doi.org/10.1021/JA512745Y.

(14) Kaczmarek, A. M.; Liu, J.; Laforce, B.; Vincze, L.; Hecke, K. Van; Deun, R. Van. Cryogenic Luminescent Thermometers Based on Multinuclear Eu^{3+}/Tb^{3+} Mixed Lanthanide Polyoxometalates. *Dalton Trans.* **2017**, *46* (18), 5781–5785. https://doi.org/10.1039/C7DT01058C.

(15) Liu, X.; Akerboom, S.; Jong, M. de; Mutikainen, I.; Tanase, S.; Meijerink, A.; Bouwman, E. Mixed-Lanthanoid Metal–Organic Framework for Ratiometric Cryogenic Temperature Sensing. *Inorg.*

Chem. **2015**, *54* (23), 11323–11329. https://doi.org/10.1021/ACS.INORGCHEM.5B01924.

(16) D'Vries, R. F.; Álvarez-García, S.; Snejko, N.; Bausá, L. E.; Gutiérrez-Puebla, E.; De Andrés, A.; Monge, M. Á. Multimetal Rare Earth MOFs for Lighting and Thermometry: Tailoring Color and Optimal Temperature Range through Enhanced Disulfobenzoic Triplet Phosphorescence. *J. Mater. Chem. C* **2013**, *1* (39), 6316–6324. https://doi.org/10.1039/c3tc30858h.

(17) Wang, Z.; Ananias, D.; Carné-Sánchez, A.; Brites, C. D. S.; Imaz, I.; Maspoch, D.; Rocha, J.; Carlos, L. D. Lanthanide-Organic Framework Nanothermometers Prepared by Spray-Drying. *Adv. Funct. Mater.* **2015**, *25* (19), 2824–2830. https://doi.org/10.1002/adfm.201500518.

(18) Ren, M.; Brites, C. D. S.; Bao, S.-S.; Ferreira, R. A. S.; Zheng, L.-M.; Carlos, L. D. A Cryogenic Luminescent Ratiometric Thermometer Based on a Lanthanide Phosphonate Dimer. *J. Mater. Chem. C* **2015**, *3* (33), 8480–8484. https://doi.org/10.1039/C5TC01468A.

(19) Ananias, D.; Brites, C. D. S.; Carlos, L. D.; Rocha, J. Cryogenic Nanothermometer Based on the MIL-103(Tb,Eu) Metal-Organic Framework. *Eur. J. Inorg. Chem.* **2016**, *2016* (13–14), 1967–1971. https://doi.org/10.1002/ejic.201501195.

(20) N'Dala-Louika, I.; Ananias, D.; Latouche, C.; Dessapt, R.; Carlos, L. D.; Serier-Brault, H. Ratiometric Mixed Eu-Tb Metal-Organic Framework as a New Cryogenic Luminescent Thermometer. *J. Mater. Chem. C* **2017**, *5* (42), 10933–10937. https://doi.org/10.1039/c7tc03223d.

(21) Ananias, D.; Mongis, C. M.; Carlos, L. D.; Rocha, J. Cryogenic Luminescent Ratiometric Thermometers Based on Tetragonal Na[LnSiO4]·xNaOH (Ln = Gd, Tb, Eu; x ≈ 0.2). *Eur. J. Inorg. Chem.* **2020**, *2020* (19), 1852–1859. https://doi.org/10.1002/EJIC.202000057.

(22) Kökçam-Demir, Ü.; Goldman, A.; Esrafili, L.; Gharib, M.; Morsali, A.; Weingart, O.; Janiak, C. Coordinatively Unsaturated Metal Sites (Open Metal Sites) in Metal-Organic Frameworks: Design and Applications. *Chem. Soc. Rev.* **2020**, *49* (9), 2751–2798. https://doi.org/10.1039/c9cs00609e.

(23) Ghanbari, T.; Abnisa, F.; Wan Daud, W. M. A. A Review on Production of Metal Organic Frameworks (MOF) for CO_2 Adsorption. *Sci. Total Environ.* **2020**, *707*, 135090. https://doi.org/10.1016/J.SCITOTENV.2019.135090.

(24) Herdes, C.; Sarkisov, L. Computer Simulation of Volatile Organic Compound Adsorption in Atomistic Models of Molecularly Imprinted Polymers. *Langmuir* **2009**, *25* (9), 5352–5359. https://doi.org/10.1021/LA804168B.

(25) Sarkisov, L.; Harrison, A. Computational Structure Characterisation Tools in Application to Ordered and Disordered Porous Materials. *Mol. Simul.* **2011**, *37* (15), 1248–1257. https://doi.org/10.1080/08927022.2011.592832.
